# Osteoarthritis burden across 204 countries and territories during 1990 to 2021: Insights from the Global Burden of Disease Study 2021

**DOI:** 10.1097/MD.0000000000046612

**Published:** 2025-12-19

**Authors:** Tao Zeng, Wei-Qi Liu, Yao-Hui Zhou, Shu-Feng Ye, Zi-Liang Chen

**Affiliations:** a Zhongshan Hospital of Traditional Chinese Medicine Affiliated to Guangzhou University of Traditional Chinese Medicine, Zhongshan, Guangdong Province, China; b Zhongshan Hospital of Traditional Chinese Medicine, Zhongshan, Guangdong Province, China.

**Keywords:** disability-adjusted life years, Global Burden of Disease, osteoarthritis, risk factors, Sociodemographic Index

## Abstract

This study provides a comprehensive assessment of the global, regional, and national burden of osteoarthritis from 1990 to 2021, based on data from the Global Burden of Disease Study 2021. We analyzed prevalence, incidence, and disability-adjusted life years (DALYs) across 204 countries and territories, stratified by age, sex, and Sociodemographic Index (SDI). In 2021, osteoarthritis affected an estimated 606.9 million people globally, with 46.6 million new cases and 21.3 million DALYs. Age-standardized prevalence, incidence, and DALY rates increased by 8.9%, 9.2%, and 9.7% respectively compared to 1990. The highest prevalence was observed in high-SDI countries such as South Korea, Brunei, and Singapore. Notably, the burden was consistently higher in women and increased with age. High body mass index and metabolic risks were the leading contributors to DALYs. These findings highlight osteoarthritis as a growing public health concern, particularly among older adults and in high-SDI regions, underscoring the need for targeted prevention and management strategies.

## 1. Introduction

Osteoarthritis is a prevalent chronic musculoskeletal disorder characterized by progressive joint degeneration and is a primary cause of chronic pain and disability among adults.^[1–3]^ Despite being preventable, osteoarthritis remains incurable once it has developed. However, the implementation of effective self-management strategies can significantly alleviate the disease burden and enhance overall quality of life.^[4]^ The 2015 World Health Organization Global Report on Ageing and Health identifies osteoarthritis as a principal contributor to disability among adults aged 60 years and older.^[5]^ From 1990 to 2019, the age-standardized disability-adjusted life year (DALY) rate for global diseases decreased by 29%, signaling a notable improvement in overall health. However, the age-standardized DALY rate for osteoarthritis increased by 10% during the same period.^[6]^ Osteoarthritis is a common joint disease, closely related to aging, obesity, overuse, or injury of joints.^[7,8]^ Clinical manifestations of osteoarthritis, such as joint pain, stiffness, swelling, and dyspraxia, negatively affecting the patient’s quality of life.^[9]^ Osteoarthritis can influence the prognosis of other diseases, such as gastrointestinal disorders, mental health conditions, diabetes, and Alzheimer disease.^[10–12]^

Presently, the management of osteoarthritis involves a combination of approaches. These include exercise and various physical therapy methods, the use of supportive tools like canes or splints, implementing self-management educational initiatives, administering pain-relieving medications, and surgical options such as joint replacement.^[13]^ Established modifiable risk factors for osteoarthritis include trauma history, gender (female), high body mass index, and metabolism.^[14]^ To ensure the precision and effectiveness of preventive and interventional measures, it is essential to quantify the distribution patterns of osteoarthritis prevalence and disease burden across different genders, age groups, and geographical regions. The Global Burden of Disease (GBD) Study comprehensively assesses health loss for 371 diseases and injuries by age, sex, year, and geographical location, enabling the comparison of burden across disparate diseases.^[15]^

The latest GBD analysis, covering the period from 1990 to 2020, has provided valuable insights into the prevalence and disease burden of osteoarthritis.^[16]^ However, previous analyses have not conducted stratified evaluations of osteoarthritis across different Sociodemographic Index (SDI) regions, genders, and age groups. Given the importance of understanding the nuanced variations in disease burden across diverse populations, it is now imperative to update the data and conduct a comprehensive and systematic evaluation of the disease burden of osteoarthritis. In this study, we report the prevalence, incidence, and DALYs associated with osteoarthritis by age, sex, and SDI in 204 countries and territories from 1990 to 2021.

## 2. Methods

Osteoarthritis, as defined by GBD 2021, is a chronic condition affecting joints, diagnosed with radiographic evidence and symptoms of pain lasting at least 1 month within the past year. The GBD 2021 study estimated the burden of 371 diseases and injuries and 88 risk factors from 1990 to 2021 in 204 countries and territories and 21 regions.^[15]^ Comprehensive accounts of the employed methodologies have been documented, with both lethal and non-lethal projections made available.^[15,17,18]^ (https://vizhub.healthdata.org/gbd-compare/ and https://vizhub.healthdata.org/gbd-results/).

The data obtained from the GBD 2021 for osteoarthritis included the prevalence, incidence and DALYs of 204 countries and territories and 21 regions. In the GBD 2021 study, osteoarthritis is characterized by symptomatic osteoarthritis affecting the hip, knee, hand, and other joints. This condition is diagnosed through radiographic evidence using the Kellgren–Lawrence grading system, which includes grade 2 (definite osteophytes and joint space narrowing) or grades 3 to 4 (multiple moderate osteophytes, significant joint space narrowing with deformity and sclerosis). Additionally, individuals must experience pain for at least 1 month within the preceding 12 months.^[19]^ Disability weight values spanning from 0 (representing full health) to 1 (signifying death) were utilized to show the extent of health loss linked to osteoarthritis. Disability weights in GBD 2021 were derived from data gathered through publicly accessible online surveys.^[20,21]^ DALYs for osteoarthritis were calculated by combining the years of life lost due to premature mortality and the years lived with disability, which were obtained by multiplying the prevalence of each sequela category by the corresponding disability weight.

The SDI is a composite measure that encompasses a lagging distribution of per capita income (which is the gross domestic product per capita smoothed over the previous decade), the average years of schooling for the population aged 15 and above, and a total fertility rate of less than 25. The SDI varies between 0, symbolizing less developed, and 1, representing most developed. The data sources for estimating the burden of osteoarthritis in various countries and regions can be accessed through the GBD 2021 Data Input Sources Tool at this link: (https://vizhub.healthdata.org/gbd-results/).

For the purpose of age-standardization, we utilized the world standard population as defined by the GBD 2021 study. This standard population is based on the age distribution of the world’s population in the year 2000 and is kept constant over time for comparison purposes. The age-standardized rates were calculated using this standard population to adjust the prevalence, incidence, and DALYs to a common age structure, allowing for more equitable comparisons across different countries and regions.

Although the GBD 2021 study documented osteoarthritis data spanning from 1990 to 2021, we utilized the R programming language (The R Foundation for statistical Computing, Vienna, Austria, https://www.r-project.org/) as a tool to create graphs. The aim was to display the prevalence, incidence and DALYs of osteoarthritis, and to compute the correlation coefficient between the age-standardized DALYs rate and the SDI across regions, countries and territories. During the study period, the number of individuals living with osteoarthritis and the age-standardized prevalence were reported along with 95% uncertainty intervals (UIs).

In our analysis, we utilized the 95% UIs provided directly by the GBD 2021 study for all estimates of prevalence, incidence, and DALYs. These intervals were calculated using advanced statistical modeling techniques and are already included in the downloaded dataset. For instances where custom calculations were necessary, we employed the formula: CI = estimate ± (*z*/times SE), using a *z*-score of 1.96 for a 95% confidence level.

To address potential missing data in our analysis, we employed several strategies. First, we assessed the extent of missing data across all variables. For variables with minimal missing data (<5%), we used the available data without imputation. For variables with a higher percentage of missing data, we utilized multiple imputation techniques to estimate the missing values. Specifically, we applied the Last Observation Carried Forward method, which assumes that the last observation carries over to the next time point. Additionally, we considered the use of regression-based imputation methods for more extensive missing data scenarios. All analyses were conducted on both the original dataset and the imputed dataset to ensure the robustness of our findings.

The R programming language was utilized to create visual representations of the calculated crude and age-standardized prevalence, incidence rates, and DALYs spanning from 1990 to 2021. These visualizations were generated both in aggregate and disaggregated forms, with the latter categorized by age groups, gender, and SDI quintiles.

Data extraction for this study was performed on April 28th. To increase the reproducibility of our study, the analysis code is available upon request. The code, written in R, includes all necessary steps to process the data from the GBD 2021 study and perform the statistical analyses as described in our methods.

## 3. Results

### 3.1. Global level

The research indicated that the number of existing osteoarthritis cases was approximately 607 million (with a 95% UIs ranging from 537.9–670.5 million). The age-standardized prevalence rate was estimated at 6967.3 per 100,000 population (95% UI from 6180.7–7686.1). Compared to the year 1990, there was a 9.0% rise in prevalence by 2021 (the 95% UI was between 8.4% and 9.5%). Globally, osteoarthritis was responsible for around 46.6 million new cases (with a 95% UI ranging from 41.0–52.0 million) in the timeframe examined, translating to an age-standardized incidence rate of 535.0 (95% UI 472.4–592.0). This rate showed a rise of 9.2% (95% UI 8.5–9.9%) from 1990 to 2021, as indicated in Table 1. In terms of DALYs at a worldwide level, osteoarthritis accounted for nearly 21.3 million DALYs (95% UI 10.2–42.9 million), corresponding to an age-standardized DALY rate of 244.5 (95% UI 117.1–493.1) per 100,000 population. This age-standardized DALY rate experienced an increase of 9.7% (95% UI 9.1–10.4%) since 1990, as documented in Table 1.

**Table 1:** Prevalent cases, incidence, and disability adjusted life years (DALYs) for osteoarthritis in 2021, and percentage change in age standardised rates (ASRs) per 100000, by Global Burden of Disease region, from 1990 to 2021 (generated from data available at https://vizhub.healthdata.org/gbd-results/) (95% UI = 95% uncertainty intervals).

	Prevalence (95% UI)			Incidence (95% UI)			DALYs (95% UI)		
	No. (in millions) (95% UI)	ASRs per 100,000 (95% UI)	Percentage change in ASRs from 1990 to 2021	No. (in thousands) (95% UI)	ASRs per 100,000 (95% UI)	Percentage change in ASRs from 1990 to 2021	No. (in thousands) (95% UI)	ASRs per 100,000 (95% UI)	Percentage change in ASRs from 1990 to 2021
Global	607 (537.9–670.5)	6967.3 (6180.7–7686.1)	9 (8.4–9.5)	46632.1 (41122.1–51644.4)	535 (472.4–592)	9.2 (8.5–9.9)	21304.6 (10189.2–42935.4)	244.5 (117.1–493.1)	9.7 (9.1–10.4)
High-income Asia Pacific	34.6 (31.1–38)	8608.6 (7674.1–9485.2)	6.6 (5.4–8)	2189.4 (1958.1–2413.2)	682.1 (606.1–752.8)	6.4 (5–7.7)	1276.8 (611.8–2579.7)	315 (150.5–636.8)	8.2 (6.7–9.8)
High-income North America	51.7 (46.3–57.3)	8421.6 (7535–9282)	5.4 (4.5–6.2)	3457.1 (3062.8–3850.8)	646.4 (572.3–715.4)	6.7 (5.8–7.6)	1857.8 (900.4–3761.7)	300.9 (144.9–607)	5.1 (4.1–6.3)
Western Europe	59.6 (53.8–66)	7113.4 (6407.1–7867.1)	5.6 (4.8–6.5)	3918.9 (3498.4–4363.2)	557.7 (497.3–618.5)	6.9 (6–8.1)	2131.3 (1038.4–4287.3)	253.6 (123.1–510.6)	6.3 (5.2–7.5)
Australasia	3.9 (3.5–4.3)	7917.6 (7098.4–8735.7)	10 (7.6–12.9)	269.5 (239.4–301.2)	620.1 (550.8–686.5)	11.6 (8.6–14.7)	140.9 (69.5–286.9)	283.4 (139.2–578)	11.4 (8–14.6)
Andean Latin America	4.4 (3.9–4.9)	7370.4 (6552.1–8123.1)	11.6 (9.6–13.8)	363.4 (319.7–402.5)	578.2 (511.3–641.1)	11.3 (8.9–13.7)	156.7 (75.1–316.2)	260.9 (125.2–526.8)	12.5 (10.2–15)
Tropical Latin America	19.4 (17.2–21.5)	7424.7 (6582.8–8241)	12.4 (11.3–13.6)	1568.5 (1385.1–1733.2)	589.1 (521.4–650.6)	11.7 (10.4–12.9)	678.6 (325.6–1368.8)	259.9 (124.7–524.6)	13.9 (12.6–15.4)
Central Latin America	19.2 (17–21.1)	7499.5 (6635.4–8259.9)	12.6 (11.3–14)	1561.6 (1379.2–1731.3)	589.5 (521.5–652.5)	11.7 (10.4–13)	676.7 (322.6–1368.4)	264.6 (126.1–535.6)	14.2 (12.8–15.8)
Southern Latin America	6.5 (5.9–7.2)	7669.2 (6896.5–8466.3)	9.5 (7.5–11.8)	483.9 (431.1–536.6)	596.3 (530.4–660.4)	10.4 (7.9–13)	233.6 (112.2–468.6)	273.5 (131.2–548.7)	10.3 (7.7–12.9)
Caribbean	3.9 (3.4–4.3)	7134.6 (6327.3–7876.6)	9.5 (7.9–11.2)	296.2 (262.5–330.7)	555.8 (493.2–617.5)	8.3 (6.7–10.1)	135.6 (64.9–274.7)	251.1 (120.1–508.3)	9.9 (8–11.8)
Central Europe	14.5 (12.8–16.2)	6948.5 (6129.2–7752.7)	10.7 (9.6–11.8)	960.1 (849.4–1070.3)	522.1 (460.8–580.4)	10.1 (9–11.2)	514.4 (248.1–1044.3)	245.4 (117.6–496.2)	12.2 (10.9–13.6)
Eastern Europe	27.1 (23.8–30.4)	7906.1 (6954–8880.1)	4.8 (3.7–5.9)	1833 (1612.6–2058.2)	585 (515.2–651.4)	6.3 (5.2–7.2)	965.6 (465.1–1951.7)	280.8 (134.4–567)	5.6 (4.2–6.9)
Central Asia	6 (5.2–6.9)	7034.9 (6120.1–8010.1)	14.5 (12.4–16.7)	475.9 (413.1–536)	504.5 (442.2–565.8)	13.4 (11.4–15.5)	212.4 (101.7–425.6)	249.3 (119.6–500.6)	15.5 (13.1–17.9)
North Africa and Middle East	30.5 (27.1–33.7)	6265.2 (5572.9–6946.2)	16.8 (15.2–18.4)	2730.8 (2402.8–3045)	488.3 (433.7–542.3)	15.1 (13.5–16.5)	1049.9 (503.1–2115.5)	215.9 (103.4–437.6)	17.7 (15.9–19.6)
South Asia	96.5 (85.6–106.7)	6326.1 (5612.4–7009.6)	17 (15.3–18.7)	8220.4 (7242–9115.9)	495 (436.6–548)	14.9 (13.5–16.5)	3311.2 (1583.9–6656.9)	216.9 (104–438)	19.2 (17.3–21.3)
Southeast Asia	39.2 (34.6–43.6)	5675.8 (5001.8–6320.9)	18.3 (16.3–20.3)	3261.5 (2872.4–3641.1)	437.1 (386.1–485)	16.2 (14.5–18.1)	1357.6 (645.2–2713.2)	196.2 (93.6–393.4)	20.1 (17.8–22.5)
East Asia	158.3 (139.5–176.8)	7036.1 (6216.3–7835.8)	14.3 (12.1–16.4)	12051.7 (10562.1–13556.2)	554.5 (486.9–619.4)	13.8 (11.9–15.9)	5518 (2633.5–11061.5)	245 (117.4–492.4)	16.1 (13.8–18.6)
Oceania	0.5 (0.5–0.6)	6196.5 (5474.5–6895)	9.9 (7.5–12.3)	47.7 (41.8–53.4)	481 (423.1–536.4)	8.9 (6.2–11.4)	17.5 (8.5–34.9)	212.9 (103–428.5)	12.1 (10.9–13.2)
Western Sub-Saharan Africa	12.8 (11.4–14.2)	6075.8 (5385.7–6757.3)	10.6 (9.6–11.6)	1226.6 (1078–1369)	483.8 (427.1–536.7)	10.1 (9.1–11)	442.1 (212.4–888.9)	210.1 (101.1–424.7)	12.1 (10.9–13.2)
Eastern Sub-Saharan Africa	10.4 (9.3–11.6)	5830 (5160.6–6476.6)	14 (11.9–15.9)	1001 (885.1–1115.5)	461 (407.4–509.9)	12 (10.4–13.5)	358.2 (171.7–719.7)	201 (96.1–405.3)	15.8 (13.6–18)
Central Sub-Saharan Africa	3.5 (3.1–3.9)	5940.5 (5268.3–6589.5)	5.6 (3.4–7.6)	339.7 (298.4–378)	463.1 (409.7–515.2)	4.8 (2.4–7.2)	120.7 (57–241.7)	204.3 (97.8–412.2)	6.8 (4.2–9.3)
Southern Sub-Saharan Africa	4.3 (3.8–4.8)	7161.2 (6333.3–7951.3)	9.2 (8.1–10.3)	375.2 (330.2–417.3)	557.2 (493.5–618.2)	8.6 (7.3–9.7)	149 (71.6–298.1)	249.5 (120.4–500.6)	8.9 (7.5–10.4)

### 3.2. Regional level

In 2021, the age-standardized prevalence of osteoarthritis per 100,000 individuals was highest at the regional level in High-income Asia Pacific, with a figure of 8608.6 (95% UI 7674.1–9485.2). It was also notably high in High-income North America (8421.6, 95% UI 7535.0–9282.0), Australasia (7917.6, 95% UI 7098.4–8735.7), Australasia (7917.6, 95% UI 7098.4–8735.7), and Eastern Europe (7906.1, 95% UI 6954–8880.1). On the other hand, Southeast Asia (5675.8, 95% UI 5001.8–6320.9), Eastern Sub-Saharan Africa (5830.0, 95% UI 5160.6–6476.6), Central Sub-Saharan Africa (5940.5, 95% UI 5268.3–6589.5), and Western Sub-Saharan Africa (6075.8, 95% UI 5385.7–6757.3) exhibited the lowest age-standardized prevalence estimates, as shown in Table 1.

The regions with the highest age-standardized osteoarthritis incidence rates were High-income Asia Pacific (682.1, 95% UI 606.1–752.8), High-income North America (646.4, 95% UI 572.3–715.4), Australasia (620.1, 95% UI 550.8–686.5), and Southern Latin America (596.3 95% UI 530.4–660.4). Conversely, Southeast Asia (437.1, 95% UI 386.1–485.0), Eastern Sub-Saharan Africa (461.0, 95% UI 407.4–509.9), Central Sub-Saharan Africa (463.1, 95% UI 409.7–515.2), and East Asia (481, 95% UI 423.1–536.4) had the lowest incidence rates, as detailed in Table 1.

In 2021, the highest age-standardized DALY rates were observed in High-income Asia Pacific (315.0, 95% UI 150.5–636.8), High-income North America (300.9, 95% UI 144.9–607.0), Australasia (283.4, 95% UI 139.2–578.0), and Eastern Europe (280.8, 95% UI 134–567.0). In contrast, Southeast Asia (196.2, 95% UI 93.6–393.4), Eastern Sub-Saharan Africa (201.0, 95% UI 96.1–405.3), Central Sub-Saharan Africa (204.3, 95% UI 97.8–412.2), and Western Sub-Saharan Africa (210.1, 95% UI 101.1–424.7) had the lowest age-standardized DALY rates, as indicated in Table 1.

Between 1990 and 2021, the age-standardized prevalence estimates underwent varying percentage changes across different GBD 2021 regions. An upward trend was observed in all regions. Notably, Southeast Asia experienced the most significant increase, with a rise of 18.3% (95% UI 16.3–20.3%). South Asia, North Africa, and the Middle East and Central Asia also had substantial increases, with respective rises of 17.0% (95% UI 15.3–18.7%), 16.8% (95% UI 15.2–18.4%), and 14.5% (95% UI 12.4–16.7%) (Table 1).

From 1990 and 2021, the percentage changes in age-standardized incidence and DALY estimates increased across all GBD 2021 regions. The top 3 increases in age-standardized incidence percentage changes were in Southeast Asia (16.2%, 95% UI 14.5–18.1%), North Africa and the Middle East (15.1%, 95% UI 13.5–16.5%), and South Asia (14.9%, 95% UI 13.5–16.5%), respectively. Moreover, these 3 regions also topped the list in terms of percentage changes in age-standardized DALY rates (Table 1).

### 3.3. National level

**Figure 1.**
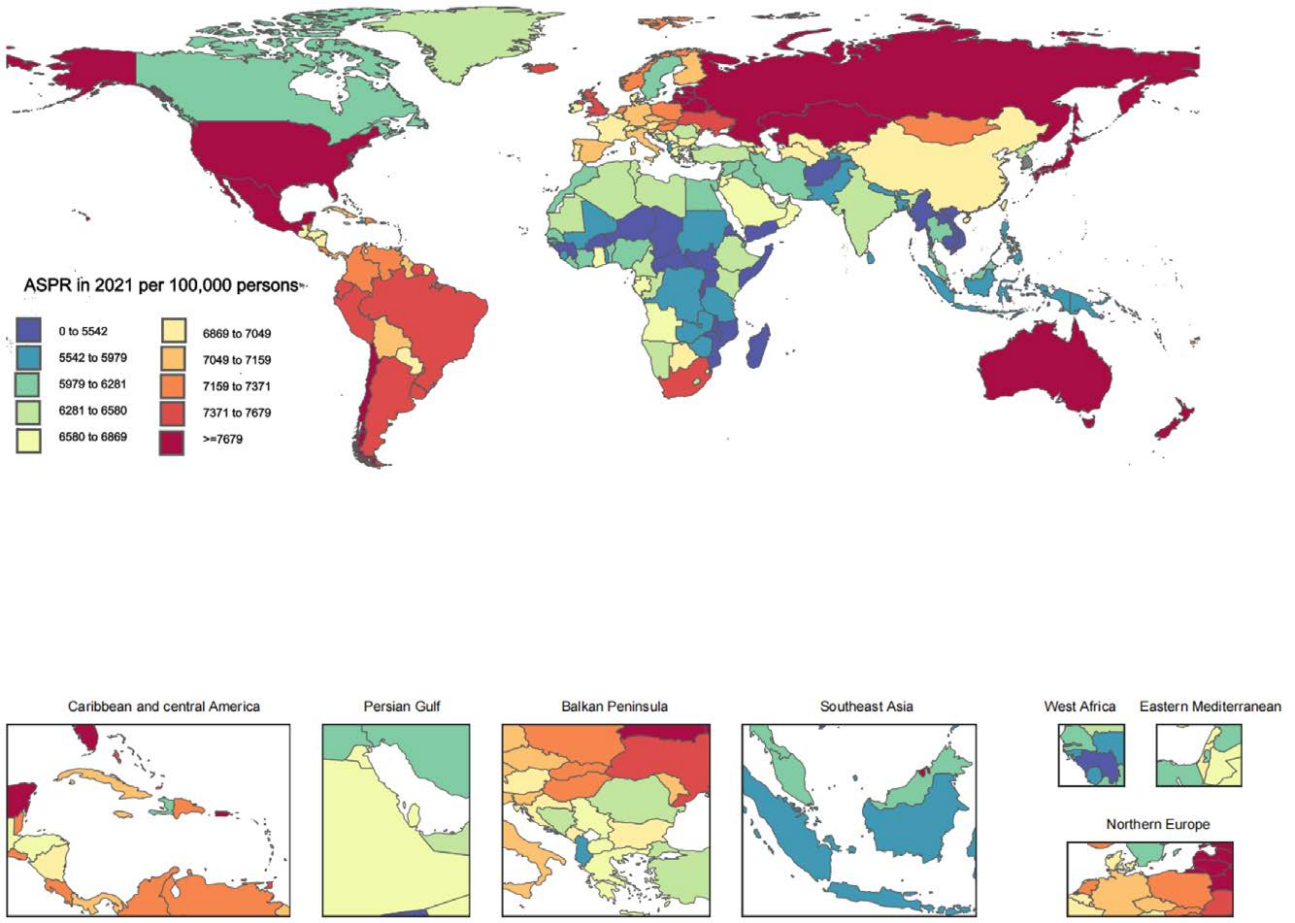
Age-standardized prevalence estimates of osteoarthritis in 2021 at national level.

In 2021, the prevalence of osteoarthritis showed substantial variation across the globe, with rates ranging from 5051 (95% UI 4487.3–5638.6) to 8997.4 cases per 100,000 individuals (95% UI 8083–9897.8). The Republic of Korea topped the list with the highest prevalence at 8997.4 (95% UI 8083.0–9897.8), followed by Brunei Darussalam at 8815.6 (95% UI 7892.9–9708.1), and Singapore at 8795.6 (95% UI 7834.8–9662.1). On the opposite end, Madagascar, Cambodia, and Burundi had the lowest rates, with 5051 (95% UI 4691.9–5937.6), 5073.2 (95% UI 4526.9–5661.5), and 5090.1 (95% UI 4515.5–5674.7) cases per 100,000 individuals, respectively. From 1990 to 2021, the age-standardized prevalence generally increased, with the Democratic Republic of the Congo showing the most significant rise at 35.1% (95% UI 29.7–40.6%), accompanied by substantial increases in Mongolia (28.1%, 95% UI 22.9–33.8%) and Ethiopia (23.3%, 95% UI 18.9–27.4%). The Netherlands, Russian Federation, and Burundi experienced minimal growth, with increases of 2.5% (95% UI −1.8% to 6.8%), 2.0% (95% UI 0.5–3.6%), and 2.4% (95% UI −0.0% to 5.2%), respectively. Denmark was the only country that showed a decrease, with a −3.4% change (95% UI −7.8% to 1.4%). Detailed data on these trends can be found in Table 2. Additionally, Figure 1 illustrates a choropleth map depicting the national age-standardized prevalence rates for the year 2021.

**Table 2:** Prevalent cases of osteoarthritis in 1990 and 2021 and the percentage change in the age-standardised rates (ASRs) per 100,000, by location (generated from data available from http://ghdx.healthdata.org/gbd-results-tool).

Location	1990		2021		Percentage change in the ASRs per 100,000
	No. (95% UI)	ASRs per 100,000 (95% UI)	No. (95% UI)	ASRs per 100,000 (95% UI)	
Global	256,076,700 (227,119,748–283,438,465)	6393.1 (5683.2–7059.5)	606,989,319 (537,873,608–670,519,617)	6967.3 (6180.7–7686.1)	9 (8.4 to 9.5)
Central Asia	2,890,239 (2,514,610–3,288,610)	6143.8 (5368.5–6965.5)	6,010,310 (5,207,826–6,870,321)	7034.9 (6120.1–8010.1)	14.5 (12.4 to 16.7)
Armenia	156,999 (136,161–177,816)	5647.3 (4927.1–6375.6)	296,566 (256,205–339,561)	6877.8 (5958.9–7835.8)	21.8 (18.1 to 26.5)
Azerbaijan	323,779 (279,068–372,562)	6391.4 (5543.1–7311.2)	771,551 (660,882–879,387)	7036.9 (6082.8–7974.3)	10.1 (6.2 to 13.8)
Georgia	383,420 (332,432–434,597)	6085.1 (5310.8–6875.3)	383,789 (333,863–434,177)	6585.2 (5732.7–7446.4)	8.2 (5 to 11.4)
Kazakhstan	831,970 (719,541–950,667)	6534.7 (5661.4–7420.2)	1,438,646 (1,254,327–1,650,413)	7753.2 (6780.8–8862.7)	18.6 (14.9 to 22.3)
Kyrgyzstan	182,459 (158,196–208,786)	6139.6 (5334.6–7001.2)	347,583 (298,907–399,945)	6870.5 (5921.5–7865.8)	11.9 (8.4 to 15.8)
Mongolia	60,106 (52,373–68,054)	5745 (5010–6500.2)	183,223 (157,823–212,343)	7361 (6383.1–8508.7)	28.1 (22.9 to 33.8)
Tajikistan	147,684 (128,100–167,793)	5395.3 (4710.3–6124.4)	370,402 (318,735–423,393)	5919.5 (5131.9–6713.7)	9.7 (6.4 to 13.2)
Turkmenistan	112,657 (97,731–128,117)	5900.6 (5140.5–6667.3)	297,031 (255,487–341,109)	7040.8 (6084.8–8034.7)	19.3 (15.2 to 23.7)
Uzbekistan	691,165 (600,001–784,590)	6016.3 (5236–6831.6)	1,921,521 (1,657,298–2,197,996)	6911.1 (5989–7871.8)	14.9 (11.7 to 18.4)
Central Europe	9,379,903 (827,4711–10,510,034)	6277 (5554.9–7001.9)	14,519,446 (12,794,343–16,234,191)	6948.5 (6129.2–7752.7)	10.7 (9.6 to 11.8)
Albania	106,048 (94,171–117,357)	5015.7 (4461.4–5552.5)	248,149 (218,659–276,764)	5802.1 (5134–6443.8)	15.7 (12.2 to 19.1)
Bosnia and Herzegovina	234,845 (206,726–262,202)	5527.4 (4885.8–6162.5)	391,736 (346,442–440,350)	6533.1 (5777.6–7326)	18.2 (14.6 to 21.9)
Bulgaria	791,590 (692,118–889,939)	6386.4 (5607.2–7153.2)	936,043 (826,382–1,044,846)	7017 (6194.4–7863.5)	9.9 (6.9 to 12.8)
Croatia	395,929 (347,321–445,003)	6364.1 (5617.2–7127.7)	565,409 (499,041–630,963)	6889 (6078–7714)	8.2 (5.4 to 11.1)
Czechia	907,575 (800,751–1,018,734)	6729.1 (5936.3–7547.5)	1,420,152 (1,247,133–1,591,851)	7158 (6278.5–8070)	6.4 (3.6 to 9)
Hungary	970,947 (856,018–1,089,127)	6709.1 (5925.5–7503.8)	1,310,359 (1,158,647–1,472,743)	7214.4 (6369–8126.7)	7.5 (4.8 to 10.1)
Montenegro	41,464 (36,197–46,589)	6534 (5736.1–7303.2)	67,345 (59,160–75,352)	6982 (6128.8–7825.1)	6.9 (3.9 to 9.8)
North Macedonia	113,855 (100,497–127,264)	5939.2 (5255.6–6635.7)	220,221 (194,055–247,313)	6595.4 (5821.3–7362.8)	11 (8.1 to 13.7)
Poland	2,760,678 (2,435,920–3,094,921)	6352.6 (5622.6–7107.5)	4,892,575 (4,294,493–5,479,012)	7209.4 (6359–8062.5)	13.5 (12.1 to 15)
Romania	1,664,333 (1,465,508–1,861,361)	5917.4 (5251.8–6600.8)	2,276,904 (2,014,050–2,545,520)	6495 (5751.5–7224.2)	9.8 (7.4 to 12.8)
Serbia	688,243 (601,329–772,952)	5940 (5240.9–6624.1)	1,032,459 (910,056–1,152,671)	6639.4 (5843.9–7416.3)	11.8 (8.5 to 14.7)
Slovakia	391,659 (342,919–440,888)	6633.9 (5808.7–7454.6)	659,577 (581,868–740,171)	7172.3 (6328.3–8022.9)	8.1 (5.4 to 11.2)
Slovenia	162,734 (142,849–182,922)	6626.2 (5823.7–7430.6)	287,181 (252,520–321,639)	7067 (6214.4–7901.7)	6.7 (3.4 to 9.7)
Eastern Europe	21,046,221 (18,447,721–23,746,861)	7541.1 (6611.1–8496.1)	27,107,908 (23,769,327–30,423,620)	7906.1 (6954–8880.1)	4.8 (3.7 to 5.9)
Belarus	894,797 (788,274–1,008,658)	6906.9 (6100.2–7765)	1,219,043 (1,073,903–1,368,927)	7779.4 (6836.9–8711.7)	12.6 (9.6 to 15.8)
Estonia	147,424 (129,808–165,855)	7234.7 (6372.2–8155.6)	194,934 (171,215–218,687)	8078 (7084.9–9173)	11.7 (8.9 to 14.5)
Latvia	251,241 (221,944–281,863)	7047.8 (6232.7–7903)	285,051 (251,601–319,884)	7920 (6968.3–8955.3)	12.4 (9.6 to 15.4)
Lithuania	312,086 (274,934–351,694)	6922.4 (6106.1–7812)	412,410 (363,536–464,231)	7829.5 (6905.5–8826.8)	13.1 (10.2 to 16.7)
Republic of Moldova	273,921 (241,769–305,215)	6181.7 (5461.8–6881.1)	414,222 (365,281–462,182)	7085.5 (6248.9–7948)	14.6 (11.7 to 18)
Russian Federation	14,229,933 (12,375,793–16,136,118)	7889.9 (6889.7–8937.7)	18,882,625 (16,518,692–21,233,250)	8051.3 (7065.9–9048.1)	2 (0.5 to 3.6)
Ukraine	4,936,819 (4,325,173–5,555,752)	6932.9 (6103.1–7792.6)	5,699,624 (5,010,777–6,405,477)	7553 (6637.2–8518)	8.9 (6 to 11.9)
Australasia	1,659,800 (1,490,005–1,834,682)	7195.3 (6466.7–7950.9)	3,922,514 (35,37,651–4,335,715)	7917.6 (7098.4–8735.7)	10 (7.6 to 12.9)
Australia	1,383,918 (1,243,299–1,529,471)	7193.3 (6457.1–7943.8)	3,294,937 (2,968,753–3,631,540)	7930.6 (7103.7–8774.5)	10.2 (7.3 to 13.6)
New Zealand	275,882 (247,472–305,447)	7201.5 (6453.8–7965.5)	627,577 (562,574–693,134)	7851.9 (7027.9–8662.2)	9 (6.5 to 11.9)
High-income Asia Pacific	16,572,952 (14,690,574–18,303,601)	8072 (7169.4–8905.9)	34,625,939 (31,148,250–37,978,128)	8608.6 (7674.1–9485.2)	6.6 (5.4 to 8)
Brunei Darussalam	9011 (8058–9964)	8218.4 (7341.2–9059)	34,032 (30,248–37,597)	8815.6 (7892.9–9708.1)	7.3 (4.6 to 9.9)
Japan	13,672,546 (12,142,309–15,127,790)	7955.1 (7071.7–8796.2)	25,378,142 (22,883,665–27,840,274)	8442.7 (7515–9306.5)	6.1 (4.8 to 7.6)
Republic of Korea	2,692,411 (2,400,450–2,973,647)	8639.5 (7715.1–9501.3)	8,441,324 (7,578,238–9,289,493)	8997.4 (8083–9897.8)	4.1 (1.4 to 6.9)
Singapore	1,98,983 (176,482–219,345)	8553.4 (7625.5–9400.2)	772,440 (686,704–849,720)	8795.6 (7834.8–9662.1)	2.8 (0.2 to 5.8)
High-income North America	26,834,459 (24,152,314–29,613,959)	7987.2 (7188.9–8825)	51,749,679 (46,318,473–57,318,852)	8421.6 (7535–9282)	5.4 (4.5 to 6.2)
Canada	1,824,018 (1,600,679–2,046,087)	5726.1 (5028.5–6431.2)	4,163,156 (3,679,838–4,663,101)	6228.3 (5509.3–6983.2)	8.8 (6 to 11.5)
Greenland	2090 (1831–2338)	5758.7 (5072.5–6443.7)	4610 (4034–5172)	6311.9 (5577.4–7018.6)	9.6 (6.9 to 12.8)
United States of America	25,007,735 (22,549,195–27,580,855)	8228.1 (7415.5–9087.5)	47,581,101 (42,638,857–52,684,415)	8686.6 (7789.7–9568.3)	5.6 (4.7 to 6.4)
Southern Latin America	3,248,110 (2,899,780–3,599,930)	7001.8 (6250.9–7759.1)	6,538,638 (5,891,362–7,213,429)	7669.2 (6896.5,8466.3)	9.5 (7.5 to 11.8)
Argentina	2,279,648 (2,035,022–2,531,460)	7040.5 (6291.8–7820.7)	4,194,311 (3,775,257–4,616,497)	7662.1 (6875.7,8441.6)	8.8 (5.9 to 12)
Chile	701,689 (626,387–776,446)	6879.9 (6154.6–7626.4)	1,951,586 (1,759,443–2,155,187)	7686.1 (6940.8–8490.8)	11.7 (8.8 to 14.7)
Uruguay	266,618 (236,980–296,555)	6998.3 (6221.7–7747.5)	392,381 (354,173–436,252)	7656.6 (6885.2–8513)	9.4 (6.3 to 12.3)
Western Europe	37,369,611 (33,648,956–41,316,152)	6736.7 (6071.8–7425)	59,567,389 (53,848,954–65,960,013)	7113.4 (6407.1–7867.1)	5.6 (4.8 to 6.5)
Andorra	3840 (3446–4260)	6593.3 (5912.7–7304.5)	10,784 (9709–11,942)	7051.4 (6342–7816.8)	6.9 (4.3 to 9.7)
Austria	750,067 (675,575–827,423)	6666.3 (5990.9–7356)	1,167,788 (1,058,283–1,289,389)	7020.5 (6338.2–7745.8)	5.3 (2.8 to 8.2)
Belgium	982,108 (881,171–1,084,366)	6669.1 (5978–7364.2)	1,478,913 (1,337,143–1,635,291)	7020.1 (6333.5–7766.1)	5.3 (2.7 to 8.3)
Cyprus	51,643 (46,463–57,278)	6280.4 (5647.3–6960.8)	139,030 (124,996–153,247)	6887.5 (6177.6–7614.1)	9.7 (7 to 12.4)
Denmark	543,685 (485,653–603,098)	7157.2 (6383.4–7947.9)	744,525 (669,361–823,260)	6913.9 (6189.6–7612.1)	-3.4 (-7.8 to 1.4)
Finland	461,234 (417,022–508,410)	6653.5 (5991.1–7344.4)	782,910 (706,234–867,240)	7050 (6353.5–7847.6)	6 (3.6 to 8.5)
France	5,232,168 (4,717,988–5,780,826)	6642.8 (5964.3–7337.3)	8,701,540 (7,878,885–9,534,117)	7026 (6328.5–7763.6)	5.8 (3.2 to 8.3)
Germany	8,283,522 (7,453,152–9,115,020)	6788.1 (6108.5–7478.5)	12,268,607 (11,049,242–13,609,528)	7101.5 (6375.1–7854.6)	4.6 (2 to 7.1)
Greece	936,120 (843,679–1,038,257)	6273.4 (5659–6962.7)	1,436,278 (1,297,937–1,588,190)	6841.4 (6128.1–7558.3)	9.1 (5.9 to 12.1)
Iceland	19,611 (17,550–21,782)	7152.3 (6408.1–7939.3)	40,058 (36,184–44,272)	7403.2 (6672.7–8204.4)	3.5 (-0.7 to 7.4)
Ireland	259,983 (232,119–287,443)	6567.1 (5836–7276.8)	532,943 (479,969–589,994)	7038.7 (6311.3–7817.1)	7.2 (4.5 to 9.8)
Israel	312,004 (280,035–345,863)	6608.7 (5921.6–7309.6)	821,936 (742,487–906,820)	7043 (6360–7771.7)	6.6 (4.1 to 9.1)
Italy	5,826,407 (5,210,209–6,466,884)	6764 (6076.3–7492)	9,171,839 (8,231,986–10,143,387)	7102.8 (6365.2–7858.7)	5 (4.2 to 5.9)
Luxembourg	35,959 (32,430–39,645)	6740.4 (6069.7–7447)	72,051 (64,857–79,547)	7055.1 (6350.9–7786.4)	4.7 (2.1 to 7.5)
Malta	28,418 (25,362–31,465)	6659.6 (5938.2–7376.8)	61,873 (55,817–68,505)	7071.7 (6385.7–7831.8)	6.2 (3.5 to 9)
Monaco	4352 (3922–4805)	6940.1 (6234.7–7689.5)	6228 (5607–6869)	7238.1 (6514.1–7963.9)	4.3 (1.5 to 7.1)
Netherlands	1,351,066 (1,239,207–1,478,701)	7007.1 (6414–7691.2)	2,323,332 (2,095,824–2,566,695)	7179.7 (6487.6–7948.1)	2.5 (-1.8 to 6.8)
Norway	426,725 (382,914–472,688)	6810.3 (6081.7–7529.3)	665,458 (598,230–735,909)	7194.4 (6445.1–7978.7)	5.6 (4.6 to 6.6)
Portugal	879,207 (785,026–974,590)	6419.4 (5732.2–7112.1)	1,531,035 (1,387,658–1,690,289)	6967.5 (6290.5–7707.6)	8.5 (5.8 to 11.3)
San Marino	2272 (2047–2511)	6783.3 (6108–7523.3)	4760 (4287–5294)	7145.9 (6446.1–7953)	5.3 (2.8 to 7.7)
Spain	3,489,572 (3,139,553–3,872,739)	6585.1 (5902.3–7293.3)	6,279,575 (5,668,451–6,993,098)	7067.4 (6346.7–7834.4)	7.3 (4.5 to 10.4)
Sweden	797,705 (708,550–892,346)	5721.6 (5030.1–6419.4)	1,188,698 (1,047,613–1,329,028)	6206.5 (5449.7–6982)	8.5 (5.9 to 11.4)
Switzerland	656,864 (587,682–726,269)	6659.3 (5952–7400.5)	1,131,233 (1,021,570–1,251,300)	6869.4 (6203.9–7594.4)	3.2 (0.9 to 5.8)
United Kingdom	6,004,365 (5,411,317–6,647,049)	7062.8 (6351.5–7819.5)	8,953,548 (8,063,136–9,891,755)	7557.5 (6798.4–8354.4)	7 (6.4 to 7.6)
Andean Latin America	1,383,606 (1,228,226–1,528,853)	6603 (5861.3–7291.1)	4,431,105 (3,933,346–4,885,804)	7370.4 (6552.1–8123.1)	11.6 (9.6 to 13.8)
Bolivia (Plurinational State of)	203,684 (180,658–226,608)	6196.2 (5522.7–6909.5)	666,486 (590,521–738,700)	7051.9 (6265.2–7829)	13.8 (10.4 to 17.2)
Ecuador	366,820 (326,444–403,918)	6764.8 (6025.1–7470.5)	1,246,864 (1,106,360–1,378,365)	7520 (6667.3–8311.4)	11.2 (8 to 14.3)
Peru	813,103 (720,159–903,442)	6641.2 (5895.3–7382.7)	2,517,755 (2,228,672–2,782,727)	7387.2 (6546.7–8191.6)	11.2 (8.4 to 14.3)
Caribbean	1,698,190 (1,502,561–1,877,189)	6514.8 (5762.2–7198)	3,852,892 (3,412,425–4,252,980)	7134.6 (6327.3–7876.6)	9.5 (7.9 to 11.2)
Antigua and Barbuda	3537 (3152–3900)	6937.5 (6157.1–7659.6)	8260 (7313–9226)	7428.8 (6608.4–8288.9)	7.1 (4.4 to 10.2)
Bahamas	11,361 (10,137–12,639)	7208 (6414.2–8012.8)	32,754 (28,801–36,383)	7623.8 (6709–8430.5)	5.8 (3.1 to 8.9)
Barbados	19,304 (17,253–21,345)	7125 (6331.3–7932.8)	38,371 (33,785–42,487)	7638.2 (6759.4–8450.3)	7.2 (4.3 to 10)
Belize	5980 (5314–6635)	6432.3 (5709.3–7146.3)	23,216 (20,541–25,751)	7355.9 (6544.1–8170.2)	14.4 (11.2 to 17.9)
Bermuda	4755 (4236–5250)	7528.6 (6700.9–8313.2)	9997 (8838–10,990)	7848.7 (6950.2–8646.8)	4.3 (1.7 to 7)
Cuba	659,381 (583,440–733,482)	6460.6 (5712–7181.6)	1,372,810 (1,213,433–1,525,483)	7149.5 (6341.9–7924.8)	10.7 (7.7 to 13.6)
Dominica	3754 (3341–4163)	6535.9 (5798.2–7263.4)	6093 (5388–6793)	7159 (6343.2–7960)	9.5 (6.7 to 12.8)
Dominican Republic	247,860 (219,597–275,061)	6527.8 (5770–7251.7)	737,841 (654,421–816,065)	7275.9 (6456–8027.5)	11.5 (8.5 to 14.7)
Grenada	4378 (3882–4886)	6439.7 (5694.5–7173.2)	8484 (7511–9413)	7182.7 (6385.5–7940.5)	11.5 (8.6 to 14.8)
Guyana	24,710 (21,921–27,494)	6351.6 (5626.3–7059.2)	48,017 (42,380–53,319)	7142.3 (6320.3–7924.3)	12.4 (8.8 to 15.4)
Haiti	180,843 (161,578–203,285)	5470 (4873.2–6126.8)	461,323 (408,504–515,426)	6017.1 (5337.4–6713.9)	10 (6.3 to 13.6)
Jamaica	111,740 (99,300–124,096)	6489.8 (5736.6–7193.4)	221,286 (197,000–244,089)	7129.5 (6347.4–7865.3)	9.9 (7.2 to 13.3)
Puerto Rico	267,147 (238,537–295,266)	7421.2 (6620.2–8213.1)	512,074 (458,025–565,681)	8021.7 (7104.5–8862.5)	8.1 (5 to 11)
Saint Kitts and Nevis	2441 (2170–2706)	6945.6 (6130.2–7687)	5643 (4966–6285)	7571.3 (6700.2–8384.8)	9 (6.1 to 11.7)
Saint Lucia	5545 (4925–6182)	6416 (5699.5–7143.8)	17,764 (15,747–19,674)	7248.7 (6444.2–8008.9)	13 (9.5 to 16.7)
Saint Vincent and the Grenadines	4460 (3970–4938)	6398 (5679.7–7065.6)	10,319 (9105–11,424)	7102.7 (6286.6–7848)	11 (7.7 to 14.1)
Suriname	18,173 (16,038–20,192)	6909.4 (6124.5–7648.5)	48,941 (43,093–54,494)	7492.6 (6632.3–8310.8)	8.4 (5.5 to 11.5)
Trinidad and Tobago	58,782 (52,329–65,064)	6947 (6166.4–7707.4)	145,746 (129,486–161,267)	7493.9 (6678.7–8293.2)	7.9 (5.2 to 10.9)
United States Virgin Islands	6535 (5788–7240)	7283.2 (6447–8083.5)	13,567 (12,093–14,990)	7850.6 (6983–8658.8)	7.8 (4.9 to 10.5)
Central Latin America	5,697,761 (5,042,112–6,304,605)	6661.1 (5900.5–7353.2)	19,197,614 (16,954,459–21,143,360)	7499.5 (6635.4–8259.9)	12.6 (11.3 to 14)
Colombia	1,169,852 (1,033,377–1,299,615)	6431.7 (5691.3–7130.3)	4,025,156 (3,565,880–4,466,445)	7248.4 (6425.2–8035.7)	12.7 (9.8 to 16)
Costa Rica	115,532 (102,589–127,712)	6530.1 (5782.3–7255.3)	404,928 (358,473–447,916)	7319.2 (6495.1–8094.9)	12.1 (9.2 to 15.2)
El Salvador	191,966 (169,693–213,065)	6409.2 (5654.7–7112.1)	443,375 (395,272–487,950)	7260.2 (6468.4–8001.8)	13.3 (10.3 to 16.7)
Guatemala	214,790 (190,429–238,338)	5948.6 (5272.3–6601.3)	748,843 (663,037–831,469)	6651.8 (5895.6–7375.2)	11.8 (8.9 to 15.4)
Honduras	127,604 (112,176–142,054)	6085.8 (5355–6786.1)	446,902 (397,250–497,172)	6762.8 (5991–7500.8)	11.1 (8.4 to 14.4)
Mexico	3,019,238 (2,680,553–3,343,444)	6890.6 (6125.7–7623.9)	10,208,237 (9,013,452–11,312,757)	7826.5 (6918.8–8646.8)	13.6 (12 to 15.1)
Nicaragua	95,092 (84,560–105,551)	6028.7 (5350.2–6702.7)	348,503 (308,821–386,203)	6877.4 (6091.9–7616.9)	14.1 (10.8 to 17.7)
Panama	93,920 (83,215–104,409)	6212.1 (5507.8–6917.3)	315,881 (280,593–347,772)	7127 (6334.2–7844.6)	14.7 (11.5 to 17.4)
Venezuela (Bolivarian Republic of)	669,767 (597,381–742,737)	6720.1 (5958–7442.2)	2,255,788 (1,984,531–2,494,580)	7290.9 (6435.3–8058.4)	8.5 (5.8 to 11.5)
Tropical Latin America	6,184,012 (5,480,738–6,851,449)	6604 (5863.6–7307.7)	19,391,655 (17,180,012–21,545,741)	7424.7 (6582.8–8241)	12.4 (11.3 to 13.6)
Brazil	6,033,182 (5,347,514–6,684,708)	6602.2 (5862.6–7307)	18,970,858 (16,804,902–21,077,260)	7433.2 (6589.6–8250.7)	12.6 (11.5 to 13.8)
Paraguay	150,830 (133,482–166,900)	6693.3 (5932.6–7381.7)	420,797 (371,505–467,033)	7045.7 (6224–7826.9)	5.3 (2.6 to 8.3)
North Africa and Middle East	9,337,845 (8,286,676–10,395,164)	5362.2 (4751.5–5979.2)	30,491,685 (27,064,615–33,709,288)	6265.2 (5572.9–6946.2)	16.8 (15.2 to 18.4)
Afghanistan	321,991 (283,429–361,804)	4551.5 (4037.5–5103.9)	546,859 (480,078–609,143)	5109.5 (4529.1–5687.2)	12.3 (8.6 to 15.7)
Algeria	677,465 (597,902–758,233)	5358.5 (4741.5–5992.2)	2,432,559 (2,150,381–2,697,828)	6415.3 (5660.5–7119.6)	19.7 (16 to 23.3)
Bahrain	12,686 (11,232–141,85)	6245.2 (5519.9–6944)	80,203 (70,946–90,083)	6726.3 (5940.4–7469.3)	7.7 (4.7 to 10.4)
Egypt	1,545,497 (1,357,027–1,727,343)	5381.8 (4766.1–5997.6)	4,288,856 (3,812,604–4,764,961)	6181.5 (5514.5–6898.7)	14.9 (11.2 to 18.3)
Iran (Islamic Republic of)	1,469,041 (1,295,481–1,641,746)	5447.5 (4832.4–6098.9)	5,083,977 (4,515,257–5,645,682)	6230.6 (5520.3–6928.6)	14.4 (13 to 15.8)
Iraq	462,767 (411,020–512,938)	5726.3 (5061.2–6360.2)	1,615,817 (1,436,788–1,801,751)	6212.4 (5513–6900)	8.5 (5.5 to 11.3)
Jordan	83,782 (73,940–93,469)	5845.3 (5186.4–6512.2)	560,039 (499,447–624,460)	6661 (5930.8–7365.3)	14 (11.2 to 17.3)
Kuwait	44,076 (39,167–49,291)	6195.3 (5519.6–6877.9)	256,931 (225,942–287,095)	6867.2 (6095.7–7617.2)	10.8 (7.3 to 14.2)
Lebanon	125,298 (110,890–140,500)	5585 (4968.9–6252.9)	386,963 (345,477–427,385)	6601.2 (5883.4–7300.5)	18.2 (14.6 to 21.6)
Libya	114,662 (101,658–126,780)	5860.1 (5206.2–6521.7)	381,939 (337,904–424,560)	6530.8 (5787.1–7228.2)	11.4 (8.7 to 14.2)
Morocco	795,767 (706,124–884,296)	5503.5 (4899.1–6128)	2,171,352 (1,923,170–2,402,601)	6061.4 (5386.6–6727.5)	10.1 (7 to 13.7)
Oman	41,161 (36,553–46,151)	5553.4 (4912.6–6176.1)	166,344 (148,368–186,293)	6610.8 (5877.9–7322.5)	19 (16 to 22.5)
Palestine	47,974 (42,524–53,957)	5473.5 (4856.5–6145.1)	174,021 (153,511–193,335)	6252.6 (5540.5–6945.8)	14.2 (10.7 to 17.9)
Qatar	10,014 (8796–11,369)	6220.4 (5474.1–6930.9)	108,211 (95,937–122,253)	6835.3 (6109.6–7568.1)	9.9 (6.7 to 13.3)
Saudi Arabia	358,102 (317,568–400,082)	5668 (5010–6297.6)	1,669,212 (1,468,855–1,864,087)	6748.9 (5982.7–7462.2)	19.1 (15.5 to 22.8)
Sudan	438,043 (388,820–493,519)	4649.5 (4118.9–5216.8)	1,200,374 (1,063,278–1,331,240)	5699.1 (5039.3–6361.3)	22.6 (18.3 to 26.4)
Syrian Arab Republic	287,369 (254,501–319,697)	5315.6 (4725.2–5952)	890,644 (783,273–991,866)	6178.6 (5461.6–6883.4)	16.2 (12.5 to 20.2)
Tunisia	281,097 (247,995–314,359)	5476.9 (4853.2–6104.7)	874,118 (773,391–972,642)	6374.7 (5641–7079.7)	16.4 (12.9 to 19.6)
Turkey	1,953,405 (1,735,774–2,176,900)	5451 (4840.2–6098)	6,265,937 (5,560,636–6,937,783)	6494.8 (5774.2–7194.6)	19.1 (15.6 to 22.9)
United Arab Emirates	35,269 (30,793–40,075)	5833.6 (5145.4–6506.8)	503,960 (441,962–572,572)	6385.1 (5686.3–7057.9)	9.5 (6.1 to 12.6)
Yemen	227,270 (201,431–254,488)	4516.4 (4006.3–5058.5)	804,925 (712,722–894,559)	5330.2 (4703.3–5941.5)	18 (13.9 to 21.8)
South Asia	32,454,745 (28,765,905–35,895,035)	5407 (4798.7–5985.6)	96,531,169 (85,576,494–106,691,001)	6326.1 (5612.4–7009.6)	17 (15.3 to 18.7)
Bangladesh	2,470,611 (2,192,026–2,737,063)	5088.7 (4524.3–5639.1)	8,297,327 (7,370,625–9,214,630)	5823.5 (5178.3–6468.2)	14.4 (11.2 to 17.8)
Bhutan	13,319 (11,794–14,892)	5197.3 (4636–5794.2)	37,238 (33,076–41,199)	5962.9 (5303.8–6602.7)	14.7 (11.6 to 18)
India	26,639,507 (23,639,835–29,480,349)	5507.8 (4893–6104.5)	79,214,251 (70,296,948–87,586,540)	6450.1 (5727.5–7137.4)	17.1 (15.3 to 18.9)
Nepal	477,016 (423,058–531,853)	4848.2 (4313.7–5394.9)	1,354,754 (1,202,296–1,508,078)	5637.5 (5020.5–6276.1)	16.3 (13 to 19.7)
Pakistan	2,854,292 (2,512,825–3,197,229)	4966.4 (4389.8–5580)	7,627,598 (6,679,953–8,492,197)	5854.8 (5137.3–6550.2)	17.9 (15.1 to 20.7)
East Asia	55,506,569 (48,473,139–62,109,118)	6157.5 (5425.4–6866.9)	158,285,424 (139,469,183–176,824,968)	7036.1 (6216.3–7835.8)	14.3 (12.1 to 16.4)
China	53,352,515 (46,603,087–59,685,781)	6148.9 (5417.3–6855.9)	152,848,106 (134,655,962–170,842,263)	7030.7 (6211.2–7831.7)	14.3 (12.1 to 16.5)
Democratic People’s Republic of Korea	1,049,464 (928,192–1,174,687)	6082.8 (5405.9–6773.9)	2,222,257 (1,964,539–2,482,778)	6522.4 (5756.5–7259.6)	7.2 (2.7 to 11.3)
Taiwan	1,104,590 (973,882–1,230,178)	6717.4 (5931.8–7456)	3,215,061 (2,855,622–3,595,342)	7761.1 (6900–8653)	15.5 (11.6 to 19)
Oceania	176,696 (156,978–197,593)	5637.2 (5022.9–6261.4)	508,189 (451,273–565,165)	6196.5 (5474.5–6895)	9.9 (7.5 to 12.3)
American Samoa	1737 (1537–1950)	7179.9 (6341.4–8017.7)	3972 (3516–4397)	7748.3 (6881.1–8567.1)	7.9 (4.7 to 10.8)
Cook Islands	892 (792–991)	6847.3 (6086.2–7589.9)	2010 (1775–2225)	7820.7 (6948.5–8635.6)	14.2 (10.8 to 17.4)
Fiji	24,591 (21,891–27,546)	6324.6 (5621.3–7061.4)	60,349 (53,323–67,176)	7350 (6534.7–8144.3)	16.2 (12.7 to 19.5)
Guam	5724 (5078–6419)	6899.5 (6103.1–7697.3)	16,202 (14,300–17,995)	7662.8 (6785–8492.6)	11.1 (7.6 to 14.3)
Kiribati	2482 (2203–2782)	6504.8 (5779.5–7252.5)	5506 (4883–6152)	7072.4 (6288.7–7879.5)	8.7 (5.5 to 11.8)
Marshall Islands	1053 (931–1170)	6338.8 (5581.8–7083.1)	2735 (2406–3061)	7072.5 (6218–7882.1)	11.6 (8.5 to 14.4)
Micronesia (Federated States of)	3094 (2749–3429)	6346.1 (5630.6–7059.5)	5780 (5073–6445)	7191.6 (6345.4–7963.1)	13.3 (10.5 to 16.7)
Nauru	318 (283–356)	6469.7 (5732.8–7183.7)	458 (405–513)	7401.4 (6526.7–8232.7)	14.4 (10.9 to 18.3)
Niue	147 (131–165)	6805.2 (6053.2–7651)	169 (148–187)	7654.2 (6773.9–8485.4)	12.5 (9 to 16.2)
Northern Mariana Islands	1450 (1277–1630)	6928.3 (6124.8–7717.2)	4361 (3862–4853)	7473.1 (6649.6–8256.8)	7.9 (4.8 to 11.3)
Palau	685 (610–762)	6836.9 (6071.4–7611)	1907 (1681–2139)	7584.4 (6727.2–8413.9)	10.9 (8 to 14.2)
Papua New Guinea	101,623 (89,857–113,864)	5273.9 (4678.3–5859.8)	328,342 (290,265–366,544)	5806.1 (5140.1–6474.7)	10.1 (6.4 to 13.6)
Samoa	5669 (5063–6305)	6490.5 (5805.3–7196.1)	10,897 (9617–12,172)	7229.3 (6387.2–8051)	11.4 (7.9 to 14.6)
Solomon Islands	8227 (7302–9180)	5668.8 (5030.9–6322.9)	24,552 (21,730–27,516)	6521.5 (5770.2–7279.3)	15 (11.6 to 18.6)
Tokelau	82 (73–92)	6244.1 (5532.8–6958.8)	108 (96–120)	7321.9 (6519.9–8126.7)	17.3 (13.7 to 21)
Tonga	3637 (3214–4041)	6346.1 (5623.6–7059.8)	5827 (5172–6501)	7098.5 (6312.4–7929.7)	11.9 (8.1 to 15.9)
Tuvalu	427 (378–476)	6213.4 (5507.5–6931.8)	768 (677–861)	7164.3 (6313.5–8004.5)	15.3 (11.8 to 18.8)
Vanuatu	3587 (3152–3992)	5420.3 (4798.7–6034.7)	11,430 (10,100–12,779)	6093.1 (5398–6783.9)	12.4 (9.1 to 15.7)
Southeast Asia	12,662,656 (11,238,788–14,120,228)	4796.6 (4256.5–5377.2)	39,227,935 (34,567,607–43,609,405)	5675.8 (5001.8–6320.9)	18.3 (16.3 to 20.3)
Cambodia	204,930 (181,011–228,888)	4390.2 (3900.7–4913.7)	659,749 (587,709–739,199)	5073.2 (4526.9–5661.5)	15.6 (11.8 to 19.8)
Indonesia	4,983,948 (4,411,432–5,586,203)	4870.7 (4310.2–5475.3)	14,891,307 (13,109,592–16,672,858)	5753.5 (5067.3–6426)	18.1 (16 to 20.5)
Lao People’s Democratic Republic	94,897 (84,572–107,000)	4473.6 (3984.7–5028.5)	256,116 (225,952–284,449)	5173.4 (4569.5–5731.9)	15.6 (11.7 to 19.8)
Malaysia	525,256 (464,559–582,937)	5386.6 (4753.3–5990)	1,844,481 (1,623,467–2,046,052)	6267.1 (5520.1–6965.7)	16.3 (13 to 19.9)
Maldives	4977 (4374–5600)	5162.6 (4558.2–5779.8)	23,523 (20,544–26,423)	6113.5 (5332.1–6842.2)	18.4 (14.6 to 21.9)
Mauritius	41,942 (36,872–46,837)	5628.5 (4946.8–6267.5)	120,219 (105,220–134,579)	6427.3 (5638.2–7185.7)	14.2 (10.8 to 17.2)
Myanmar	1,077,954 (954,127–1,205,275)	4569.3 (4058.3–5124.5)	2,815,943 (2,485,901–3,143,670)	5510 (4883.6–6154.2)	20.6 (16.1 to 24.8)
Philippines	1,518,301 (1,328,785–1,694,014)	4937.2 (4334.3–5512.2)	4,905,958 (4,283,100–5,490,221)	5704.1 (4994.5–6360.8)	15.5 (14.4 to 16.9)
Seychelles	3101 (2740–3457)	5597.5 (4954.4–6239.9)	8012 (7010–8934)	6468.7 (5702.1–7176.2)	15.6 (11.8 to 19.4)
Sri Lanka	552,486 (488,604–613,796)	4907.6 (4340.6–5474)	1,575,155 (1,380,242–1,763,543)	5737.3 (5047.7–6405.7)	16.9 (12.8 to 20.9)
Thailand	1,843,097 (1,638,258–2,053,298)	4903.3 (4351.9–5480)	6,612,397 (5,856,916–7,391,249)	6000 (5325.5–6676.3)	22.4 (17.6 to 27)
Timor-Leste	13,763 (12,197–15,557)	4417 (3920.1–4966.9)	45,113 (39,773–50,301)	5191.6 (4582.5–5781.3)	17.5 (13 to 22.4)
Viet Nam	1,779,685 (1,572,373–1,987,537)	4453.3 (3942–4957.7)	5,415,247 (4,745,047–6,045,660)	5186.6 (4567.3–5777.2)	16.5 (11.8 to 21)
Central Sub-Saharan Africa	1,314,105 (1,159,131–1,464,371)	5622.8 (4965.8–6260.9)	3,517,438 (311,5894–3,914,394)	5940.5 (5268.3–6589.5)	5.6 (3.4 to 7.6)
Angola	252,574 (218,756–284,311)	6140.7 (5390.6–6872.1)	869,769 (761,428–975,501)	6744.4 (5931.1–7515.9)	9.8 (7.1 to 12.7)
Central African Republic	61,668 (54,203–68,966)	5234.3 (4629.4–5830.8)	129,290 (114,760–144,249)	5416.4 (4810.5–6077.9)	3.5 (0.3 to 6.2)
Congo	65,397 (57,773–72,901)	6008.2 (5309–6688.8)	196,135 (174,147–218,303)	6579.6 (5822.3–7269.4)	9.5 (6.7 to 12.4)
Democratic Republic of the Congo	891,752 (786,538–994,781)	5495.1 (4869.5–6140.5)	2,209,192 (1,952,824–2,450,224)	5630.4 (4986.7–6263.4)	2.5 (-0.6 to 5.2)
Equatorial Guinea	10,007 (8847–11,211)	5036.5 (4473.5–5638.1)	37,815 (33,406–42,295)	6803.4 (6010.9–7551.6)	35.1 (29.7 to 40.6)
Gabon	32,706 (28,948–36,480)	5726.8 (5087.2–6371.2)	75,237 (67,263–83,893)	6715.9 (6006–7495.8)	17.3 (13.9 to 21.1)
Eastern Sub-Saharan Africa	3,912,597 (3,470,701–4,378,272)	5113.7 (4544.3–5704.4)	10,409,572 (9,265,846–11,557,319)	5830 (5160.6–6476.6)	14 (11.9 to 15.9)
Burundi	116,072 (103,027–129,637)	4971.2 (4410–5556.2)	264,423 (233,713–296,285)	5090.1 (4515.5–5674.7)	2.4 (0 to 5.2)
Comoros	10,343 (9170–11,584)	5099.1 (4529.1–5712.8)	29,375 (25,982–32,841)	5730.4 (5090.9–6425.5)	12.4 (8.8 to 15.6)
Djibouti	7546 (6713–8440)	5098.6 (4541–5685.9)	43,543 (38,605–48,600)	6078.3 (5360.1–6782.8)	19.2 (15.5 to 22.9)
Eritrea	57,678 (51,105–64,537)	4815.4 (4277.2–5363.6)	159,726 (141,263–178,274)	5346.1 (4733.6–5981.6)	11 (7.5 to 14.5)
Ethiopia	1,040,403 (920,397–1,164,694)	5154.7 (4583.3–5784.5)	2,881,340 (2,528,598–3,201,412)	6355.2 (5595.8–7115.1)	23.3 (18.9 to 27.4)
Kenya	470,145 (416,631–523,478)	5591.6 (4952.6–6240.3)	1,600,840 (1,411,451–1,776,033)	6578.3 (5819.6–7327.8)	17.6 (15.5 to 19.7)
Madagascar	246,450 (218,907–275,235)	4777.4 (4264.7–5337.4)	618,496 (547,973–692,259)	5051 (4487.3–5638.6)	5.7 (2.9 to 8.7)
Malawi	196,014 (174,248–220,148)	4979.9 (4428.5–5585.1)	430,596 (381,851–480,035)	5536.2 (4902.5–6192.4)	11.2 (7.7 to 14.6)
Mozambique	302,315 (266,627–338,110)	4868.4 (4318.7–5423.7)	638,138 (563,558–713,790)	5413.4 (4778.3–6077.6)	11.2 (7.6 to 14.4)
Rwanda	138,831 (123,263–155,503)	4847 (4312–5421.2)	354,899 (313,940–399,398)	5300.4 (4691.9–5937.6)	9.4 (5.9 to 12.3)
Somalia	126,940 (112,767–141,973)	4936.4 (4380.8–5554.1)	339,052 (300,120–377,062)	5154.5 (4571.3–5746.8)	4.4 (1.1 to 7.4)
South Sudan	124,312 (110,890–139,114)	4833 (4315.1–5389.3)	219,211 (193,940–245,024)	5173.4 (4585.4–5772.6)	7 (3.6 to 10.6)
Uganda	319,756 (282,998–358,253)	4869.5 (4324.8–5462.9)	846,302 (748,400–943,277)	5411.1 (4799.8–6007.1)	11.1 (8 to 14.6)
United Republic of Tanzania	593,225 (524,534–660,944)	5346.7 (4755.9–5958.3)	1,545,397 (1,374,111–1,718,530)	5701.5 (5083.8–6350.5)	6.6 (3.9 to 9.5)
Zambia	159,770 (141,651–177,776)	5409.1 (4790.7–6032.4)	429,174 (380,416–476,903)	5732 (5074.7–6383.2)	6 (3.3 to 9.1)
Southern Sub-Saharan Africa	1,801,904 (1,590,783–2,000,266)	6559.8 (5794.8–7289)	4,289,179 (3,773,086–4,755,346)	7161.2 (6333.3–7951.3)	9.2 (8.1 to 10.3)
Botswana	33,412 (29,776–37,408)	5823 (5193.8–6519.6)	109,840 (97,517–122,269)	6937.4 (6147.1–7710.6)	19.1 (15.7 to 22.5)
Eswatini	17,749 (15,782–19,682)	5923.7 (5253.8–6600.1)	40,512 (36,025–45,099)	6853.4 (6098.8–7609.4)	15.7 (11.9 to 19.5)
Lesotho	46,614 (41,366–52,080)	5447 (4831.7–6051.3)	71,756 (63,484–79,522)	6488.5 (5745.1–7198.4)	19.1 (15.1 to 23.1)
Namibia	36,282 (32,170–40,576)	5491.1 (4865–6137.2)	91,169 (81,462–101,415)	6282.6 (5591.3–6986.7)	14.4 (10.9 to 17.8)
South Africa	144,3350 (127,1319–1,603,519)	6897.7 (6089.4–7678.1)	3,566,080 (3,135,481–3,969,604)	7437.8 (6576.7–8268.6)	7.8 (6.7 to 9.1)
Zimbabwe	224,498 (199,247–250,423)	5372.6 (4786.1–6015.1)	409,821 (362,490–457,874)	5598.2 (4951.9–6252.7)	4.2 (1.2 to 7.3)
Western Sub-Saharan Africa	4,944,718 (4,378,452–5,533,517)	5494.2 (4872–6124.7)	12,813,642 (11,400,709–14,172,790)	6075.8 (5385.7–6757.3)	10.6 (9.6 to 11.6)
Benin	106,754 (94,727–119,331)	5318.9 (4714.4–5935.7)	343,780 (306,792–381,590)	6253.8 (5563.7–6986.8)	17.6 (13.8 to 21.3)
Burkina Faso	217,159 (192,068–243,495)	4913.1 (4360.6–5506.1)	521,317 (463,358–586,311)	5374 (4778.2–6014.4)	9.4 (6.4 to 13.1)
Cabo Verde	11,781 (10,398–13,212)	5361.9 (4732.9–6021.1)	29,551 (26,139–32,888)	6384.6 (5666.9–7115.5)	19.1 (15.3 to 23)
Cameroon	259,289 (230,455–290,264)	5590.6 (4980.4–6247.2)	852,479 (762,206–940,131)	6290.7 (5604.2–6947.5)	12.5 (9.1 to 15.9)
Chad	139,180 (123,430–155,559)	4928.7 (4386.6–5501)	318,880 (282,304–358,854)	5195.5 (4610.4–5833.6)	5.4 (2.2 to 8.7)
Côte d’Ivoire	230,694 (205,328–256,963)	5375.5 (4761.3–5987.4)	733,425 (653,903–817,876)	5989.8 (5296.4–6648.8)	11.4 (8.3 to 14.5)
Gambia	19,411 (17,255–21,783)	5354.1 (4758.3–5999.4)	64,114 (56,832–70,970)	6266.3 (5546.6–6941.8)	17 (13.1 to 20.7)
Ghana	411,219 (361,888–458,706)	6293.9 (5570.5–7038.6)	1,183,031 (1,050,661–1,309,266)	6596.6 (5839.4–7323.1)	4.8 (1.7 to 8.4)
Guinea	167,938 (148,987–187,462)	5023.8 (4471.8–5601.2)	320,100 (284,903–358,792)	5472.9 (4862.6–6116.3)	8.9 (5.6 to 12.3)
Guinea-Bissau	20,807 (18,382–23,276)	5149.5 (4564.9–5748.6)	43,795 (38,803–48,753)	5632.2 (4996.8–6286.3)	9.4 (6.1 to 12.6)
Liberia	62,347 (55,424–69,475)	5391.3 (4784–6010.3)	144,112 (128,549–160,569)	6111 (5430.8–6772.9)	13.4 (10.2 to 16.3)
Mali	205,635 (18,0947–232,197)	5003.7 (4436.3–5626)	518,280 (460,244–576,198)	5557.5 (4935–6183.5)	11.1 (7.3 to 14.3)
Mauritania	55,961 (49,822–62,500)	5543.3 (4943.5–6184.4)	142,474 (126,133–157,303)	6313.6 (5564.2–6966.4)	13.9 (10.8 to 17.5)
Niger	143,841 (127,678–160,716)	4942.5 (4402.7–5542.9)	45,4881 (401,578–510,376)	5232.1 (4650.2–5890.5)	5.9 (2.7 to 9.4)
Nigeria	2,538,174 (2,252,321–2,836,865)	5626.7 (4985.2–6276)	6,171,577 (5,479,716–6,850,080)	6255.9 (5535.5–6973.4)	11.2 (9.9 to 12.4)
Sao Tome and Principe	3684 (3245–4140)	5685.9 (5023.1–6379)	8195 (7249–9177)	6782.5 (5994.6–7537.7)	19.3 (15.2 to 23.4)
Senegal	178,536 (158,272–200,005)	5413.8 (4796–6047.4)	487,696 (431,956–543,741)	6000.7 (5295.7–6683.9)	10.8 (7.8 to 13.9)
Sierra Leone	104,474 (92,952–117,306)	5080.1 (4527–5700)	228,399 (202,457–254,359)	5743.7 (5103.8–6394)	13.1 (9.2 to 16.6)
Togo	67,671 (60,130–75,120)	5239.9 (4654.1–5851.1)	247,408 (218,789–274,359)	5942 (5283.6–6623.7)	13.4 (9.8 to 17.6)

UI = uncertainty intervals.

**Figure 2.**
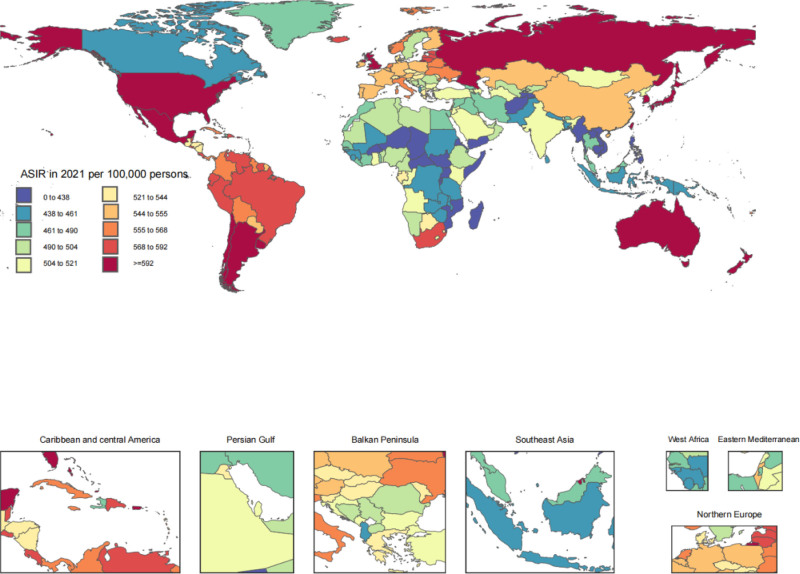
Age-standardized incidence estimates of osteoarthritis in 2021 at national level.

In 2021, the standardized incidence rates of osteoarthritis varied widely, ranging from 350.2 to 776.8 new cases per 100,000 individuals. Among the countries with the highest age-standardized incidence rates of osteoarthritis in 2021 were the Republic of Korea (701.2, 95% UI 625.4–776.8), Brunei Darussalam (686.7, 95% UI 609.4–759.0), and Singapore (685.7, 95% UI 606.5–760.5). In 1990, the rates were 665.9 (592.3–739.2), 638.1 (565.5–711), and 662.5 (583.5–735.5) for these countries. In 2021, the countries with the lowest age-standardized incidence rates of osteoarthritis were Cambodia, with a rate of 395.3 (95% UI 350.2–440.7), Timor–Leste at 400.4 (95% UI 353.0–446.0), and Viet Nam, recording 400.8 (95% UI 350.7–445.4). The most significant rises in age-standardized incidence rates of osteoarthritis from 1990 were observed in Equatorial Guinea, with an increase of 29.8% (95% UI 24.7–34.9%), Mongolia, experiencing a 22.5% rise (95% UI 17.8–26.9%), and Thailand, which saw a 20.7% increase (95% UI 16.0–25.7%). Among the 204 countries, only Denmark exhibited a slight downward trend in its age-standardized incidence rate of osteoarthritis from 1990 to 2021, with a change of −0.7% (95% UI −4.9% to 4.0%) (Table 3). Figure 2 presents a global choropleth map depicting age-standardized incidence rates at the national level for 2021.

**Table 3:** Incident cases of osteoarthritis in 1990 and 2021 and the percentage change in the age-standardised rates (ASRs) per 100,000, by location (generated from data available from http://ghdx.healthdata.org/gbd-results-tool).

Location	1990		2021		Percentage change in the ASRs per 100,000
	No. (95% UI)	ASRs per 100,000 (95% UI)	No. (95% UI)	ASRs per 100,000 (95% UI)	
Global	20,900,510 (18,467,653–23,104,316)	489.8 (433.1–541.5)	46,632,144 (41,122,053–51,644,431)	535 (472.4–592)	9.2 (8.5 to 9.9)
Central Asia	221,869 (194,411–249,130)	445 (392.6–498.5)	475,854 (413,121–535,995)	504.5 (442.2–565.8)	13.4 (11.4 to 15.5)
Armenia	12,390 (10,851–13,990)	417.8 (366.1–467.5)	19,890 (17,446–22,468)	498.4 (434–561.5)	19.3 (15 to 23.4)
Azerbaijan	25,059 (21,623–28,311)	457 (400.4–514.6)	61,653 (53,118–70,037)	507.8 (443.4–571)	11.1 (7.8 to 14.6)
Georgia	27,868 (24,387–31,363)	446.3 (394.5–501)	24,890 (21,860–27,992)	479.3 (420.9–539.3)	7.4 (4.2 to 10.6)
Kazakhstan	64,185 (56,186–72,298)	468.3 (412.4–524)	109,192 (95,014–122,963)	545.3 (477.2–610.9)	16.4 (12.9 to 20.4)
Kyrgyzstan	13,634 (11,891–15,304)	441.3 (385.5–493.9)	28,256 (24,309–32,133)	491 (425.1–552.1)	11.3 (7.9 to 14.5)
Mongolia	4849 (4251–5445)	422 (371.6–472.4)	15,754 (13,472–18,099)	517.1 (448.9–583.2)	22.5 (17.8 to 26.9)
Tajikistan	11,678 (10,187–13,143)	398.5 (351–448)	32,556 (27,991–36,814)	437.7 (382.6–489.2)	9.8 (6.5 to 13.5)
Turkmenistan	9070 (7961–10,235)	431.3 (381.1–485.9)	24,003 (20,721–27,399)	504.1 (440.6–569.6)	16.9 (13.1 to 21)
Uzbekistan	53,137 (46,259–59,857)	435.6 (382.8–491.5)	159,659 (137,466–180,475)	498.9 (434.9–559.3)	14.5 (11.1 to 18)
Central Europe	699,280 (618,328–779,466)	474.3 (419.4–525.9)	960,144 (849,389–1,070,329)	522.1 (460.8–580.4)	10.1 (9 to 11.2)
Albania	9186 (8124–10,211)	394.3 (350.8–437.5)	17,461 (15,374–19,597)	450.8 (399.8–501.8)	14.3 (11 to 17.8)
Bosnia and Herzegovina	19,585 (17,166–21,961)	425.9 (377.2–473.1)	26,327 (23,133–29,466)	492 (431.5–548.6)	15.5 (12 to 19.1)
Bulgaria	57,146 (50,342–64,003)	482.2 (426.1–535.8)	59,410 (52,594–65,848)	520.7 (461.7–577.3)	8 (4.8 to 11.2)
Croatia	29,753 (26,249–33,364)	475.8 (421–529.4)	35,691 (31,552–39,616)	514.6 (454.9–573.2)	8.2 (5.2 to 11.4)
Czechia	64,114 (56,722–72,019)	497.6 (437.9–560)	91,249 (81,004–101,782)	530.3 (467.7–594.3)	6.6 (3.8 to 9.6)
Hungary	68,521 (60,691–76,506)	500.3 (443.4–557.9)	84,065 (74,012–93,556)	534.7 (469–597.5)	6.9 (4 to 9.8)
Montenegro	3188 (2803–3555)	490.1 (430.3–544.7)	4653 (4083–5215)	520.4 (455.7–581.4)	6.2 (3.1 to 9.2)
North Macedonia	9175 (8091–10,209)	453.8 (402.1–505.5)	16,080 (14,211–18,002)	496.4 (437.4–552.5)	9.4 (6.4 to 12.4)
Poland	208,141 (183,528–232,356)	485.8 (428.3–540.9)	328,302 (290,505–366,346)	545.9 (480.2–607)	12.4 (11 to 13.8)
Romania	125,423 (110,912–140,299)	451.2 (400.3–499.8)	151,599 (133,962–168,487)	492.1 (434.7–545.1)	9.1 (6.2 to 11.9)
Serbia	53,619 (47,008–60,118)	454.1 (402.3–504.8)	68,586 (60,637–76,248)	501.3 (442.7–560.4)	10.4 (6.5 to 13.7)
Slovakia	28,455 (25,090–31,774)	493.7 (434.3–553.7)	44,551 (39,164–49,636)	530.4 (465.3–590)	7.4 (4.7 to 10.9)
Slovenia	11,790 (10,384–13,186)	491.1 (431.9–546.6)	18,195 (16,123–20,342)	525.4 (463.1–586.4)	7 (3.7 to 10.2)
Eastern Europe	1,495,247 (1,312,342–1,682,319)	550.4 (484–614.2)	183,3038 (1,612,584–2,058,220)	585 (515.2–651.4)	6.3 (5.2 to 7.2)
Belarus	63,143 (55,568–70,532)	507 (447.2–566.8)	80,484 (70,518–90,198)	564.4 (497.2–630.2)	11.3 (8.4 to 14.6)
Estonia	10,258 (9077–11,434)	526.3 (466–586.9)	11,715 (10,395–13,130)	582.9 (509.6–651)	10.8 (7.5 to 14)
Latvia	17,577 (15,584–19,601)	518 (458.9–575)	17,147 (15,282–19,092)	574.1 (507.9–640)	10.8 (7.7 to 14.4)
Lithuania	22,207 (19,616–24,958)	511.2 (452.6–572)	24,886 (21,924–27,897)	568.4 (499.9–637.7)	11.2 (7.9 to 14.6)
Republic of Moldova	21,214 (18,723–23,577)	466.9 (413.2–517.8)	28,864 (25,566–32,254)	527.5 (467.4–589.4)	13 (9.9 to 16.7)
Russian Federation	1,004,303 (881,010–1,125,128)	567.4 (498.6–635.2)	1,283,831 (1,130,169–1,446,184)	595.2 (523.1–664.6)	4.9 (3.8 to 6)
Ukraine	356,545 (312,760–401,590)	523.4 (460–582.7)	386,110 (341,241–431,370)	563.9 (496–629.1)	7.7 (4.7 to 10.9)
Australasia	124,186 (11,0781–137,553)	555.8 (493.6–617.2)	269,452 (239,374–301,169)	620.1 (550.8–686.5)	11.6 (8.6 to 14.7)
Australia	103,535 (92,499–114,683)	554.3 (492.6–615.9)	225,080 (199,821–251,382)	619.7 (549.2–687)	11.8 (8.1 to 15.5)
New Zealand	20,651 (18,415–22,849)	563.1 (500.6–625.2)	44,372 (39,438–49,395)	622 (553.4–687.7)	10.5 (7.6 to 13.7)
High-income Asia Pacific	1,345,776 (1,189,858–1,491,373)	641.2 (568.2–707.8)	2,189,403 (1,958,142–2,413,152)	682.1 (606.1–752.8)	6.4 (5 to 7.7)
Brunei Darussalam	907 (797–1004)	638.1 (565.5–711)	3204 (2818–3555)	686.7 (609.4–759)	7.6 (4.9 to 10.4)
Japan	1,083,158 (956,010–1,200,741)	633.4 (560.5–699.9)	1,510,141 (1,348,295–1,664,133)	671.4 (594.2–740.2)	6 (4.8 to 7.2)
Republic of Korea	243,702 (215,123–270,409)	665.9 (592.3–739.2)	615,472 (547,205–688,216)	701.2 (625.4–776.8)	5.3 (2.2 to 8.4)
Singapore	18,009 (15,810–20,088)	662.5 (583.5–735.5)	60,587 (53,460–67,211)	685.7 (606.5–760.5)	3.5 (0.6 to 6.9)
High-income North America	1,899,645 (1,695,714–2,093,720)	605.7 (535.7–670.6)	3,457,087 (3,062,826–3,850,766)	646.4 (572.3–715.4)	6.7 (5.8 to 7.6)
Canada	128,828 (114,244–144,138)	416 (365.5–464.6)	263,976 (232,772–297,165)	460.1 (405.5–513.8)	10.6 (7.5 to 13.5)
Greenland	192 (167–216)	421.4 (372.8–472.4)	355 (311–402)	471.2 (417–527.7)	11.8 (8.8 to 15)
United States of America	1,770,582 (1,582,306–1,951,370)	626.7 (554.5–694.1)	3,192,702 (2,830,532–3,554,075)	668.5 (591.7–739.6)	6.7 (5.7 to 7.6)
Southern Latin America	252,630 (224,153–281,234)	540.2 (478.8–600.9)	483,946 (431,133–536,648)	596.3 (530.4–660.4)	10.4 (7.9 to 13)
Argentina	175,241 (154,713–195,454)	541.8 (479.4–603.6)	311,890 (277,215–345,792)	594.8 (527.8–658.5)	9.8 (6.6 to 13.2)
Chile	58,214 (51,807–64,573)	536 (476.2–596)	145,188 (129,824–162,035)	599.6 (536.3–665.6)	11.9 (8.6 to 15.4)
Uruguay	19,164 (17,022–21,378)	540.2 (478.2–600.2)	26,842 (23,942–29,966)	595.3 (530.8–662)	10.2 (6.9 to 13.4)
Western Europe	2630,868 (2,349,667–2,928,270)	521.5 (465.7–578.7)	3,918,888 (3,498,382–4,363,204)	557.7 (497.3–618.5)	6.9 (6 to 8.1)
Andorra	308 (272–344)	511.6 (452.4–572.8)	806 (715–900)	551.9 (489.7–614.2)	7.9 (4.7 to 11.3)
Austria	51,885 (46,358–57,455)	512.7 (455.7–567)	78,649 (70,338–87,916)	548 (489.8–610.9)	6.9 (4 to 10.1)
Belgium	68,466 (60,992–76,513)	512.4 (456–570.6)	97,579 (86,703–108,987)	547.4 (487.4–611)	6.8 (3.8 to 10.1)
Cyprus	4014 (3569–4449)	489.2 (435.5–543.5)	10,306 (9254–11,403)	540 (480.8–598.4)	10.4 (7.2 to 13.7)
Denmark	36,837 (32,957–40,595)	542.8 (482–601)	48,848 (43,441–54,517)	539.2 (479.5–598.5)	-0.7 (-4.9 to 4)
Finland	33,541 (29,882–37,178)	512.5 (456.4–570.2)	49,030 (43,898–54,528)	550 (493.4–610.3)	7.3 (4.5 to 10.3)
France	365,821 (325,843–407,123)	511.4 (453.5–572)	558,962 (496,889–624,475)	547.5 (486–604.9)	7.1 (4 to 10.8)
Germany	579,625 (515,358–644,919)	520 (464.4–575.9)	787,712 (698,574–873,750)	553.7 (490.8–616.7)	6.5 (3.3 to 9.9)
Greece	68,732 (61,359–77,471)	493 (440.2–550.5)	92,595 (82,686–102,214)	538.1 (478.8–598.9)	9.1 (5.4 to 12.4)
Iceland	1419 (1270–1570)	549.7 (490.9–609.4)	2754 (2459–3050)	570.3 (509.8–631.8)	3.7 (-0.5 to 7.8)
Ireland	18,953 (16,954–20,966)	509.4 (451.6–566.4)	38,499 (34,403–42,750)	552 (492.9–614.5)	8.4 (5.3 to 11.5)
Israel	23,382 (20,991–25,917)	514.1 (457.7–573)	58,969 (52,884–65,863)	550.1 (491.2–613.3)	7 (4.1 to 9.9)
Italy	415,925 (369,369–465,184)	528.3 (469.8–586.7)	595,991 (530,002–664,509)	560.2 (498.2–622.4)	6 (5.1 to 7)
Luxembourg	2604 (2328–2913)	517.7 (462.1–579.8)	5250 (4679–5850)	549.7 (491.7–611.8)	6.2 (3.3 to 9.6)
Malta	2205 (1957–2451)	514.9 (455.6–572.9)	3974 (3570–4417)	551.3 (491.7–614)	7.1 (4 to 10.2)
Monaco	275 (246–307)	536.8 (480.5–597.8)	391 (350–437)	566.7 (505.3–629.2)	5.6 (2.7 to 8.8)
Netherlands	97,291 (87,433–107,938)	536.5 (482.4–594.2)	154,895 (137,845–173,273)	562.3 (499.3–625.5)	4.8 (0.6 to 9.1)
Norway	28,574 (25,449–31,542)	527.8 (468.9–586.6)	45,755 (40,756–510,04)	566.4 (504.4–629.3)	7.3 (6.1 to 8.7)
Portugal	64,091 (57,342–71,476)	499.4 (446.7–554.4)	99,154 (88,674–109,697)	545.2 (488.3–605)	9.2 (5.8 to 12.3)
San Marino	160 (142–178)	524.8 (466.4–586.1)	314 (280–351)	561.9 (499.9–630.4)	7.1 (4 to 10.1)
Spain	247,727 (219,689–278,142)	509.7 (452.2–570.1)	420,976 (376,269–470,250)	550.5 (490.5–613.9)	8 (4.7 to 11.6)
Sweden	54,140 (47,892–60,495)	448 (393.9–504.3)	77,849 (68,631–87,510)	490.5 (429.3–550.4)	9.5 (6.4 to 12.4)
Switzerland	46,179 (41,057–51,247)	512.8 (455.3–571.8)	76,109 (67,951–85,016)	536.3 (478.1–595.8)	4.6 (1.7 to 7.4)
United Kingdom	416,553 (371,985–462,864)	553.9 (494.5–614.7)	610,070 (544,798–679,814)	599.1 (535.3–664.8)	8.2 (7.5 to 8.8)
Andean Latin America	121,137 (107,214–134,640)	519.5 (461.5–577.7)	363,396 (319,748–402,547)	578.2 (511.3–641.1)	11.3 (8.9 to 13.7)
Bolivia (Plurinational State of)	18,351 (16,218–20,443)	491.7 (436.1–549.3)	57,030 (50,392–63,359)	555.5 (491.8–616.9)	13 (9.5 to 16.5)
Ecuador	32,268 (28,496–35,886)	534 (474.8–593.8)	100,807 (88,995–111,969)	590.7 (522.2–656)	10.6 (7.1 to 14.1)
Peru	70,517 (62,193–78,558)	520.8 (462.2–579.8)	205,559 (180,552–227,854)	578.8 (509.4–641.2)	11.1 (7.6 to 14.5)
Caribbean	139,108 (123,461–154,387)	513 (456–570.5)	296,170 (262,547–330,668)	555.8 (493.2–617.5)	8.3 (6.7 to 10.1)
Antigua and Barbuda	262 (235–290)	539.1 (479.3–598.4)	663 (581–741)	578.7 (509.8–644.1)	7.3 (4.3 to 11)
Bahamas	993 (876–1107)	560 (495.8–620.7)	2722 (2371–3044)	593.6 (522.4–658.8)	6 (2.7 to 9.5)
Barbados	1360 (1203–1509)	555.9 (490.8–620.5)	2710 (2387–3047)	595.2 (525.1–661.8)	7.1 (3.9 to 10.2)
Belize	497 (440–550)	509.9 (450.9–565.1)	2061 (1816–2294)	579.9 (515.9–644.6)	13.7 (10.4 to 17.8)
Bermuda	384 (339–427)	584.3 (515.1–649.1)	670 (593–746)	610 (538.1–674.8)	4.4 (1.6 to 7.7)
Cuba	52,466 (46,435–58,324)	511.3 (452.6–569.6)	99,435 (87,342–111,906)	561.7 (497.5–626)	9.9 (6.4 to 13)
Dominica	285 (254–316)	516.4 (456.6–573.8)	469 (414–523)	560.3 (497.8–619.9)	8.5 (5.3 to 12)
Dominican Republic	21,754 (19,212–24,251)	513.7 (455.3–568.9)	60,247 (53,094–67,341)	569.3 (503.4–635.7)	10.8 (7.7 to 14.4)
Grenada	313 (280–346)	507.1 (448.9–563.8)	682 (602–764)	559.5 (496.6–623.8)	10.3 (7 to 13.5)
Guyana	2240 (1981–2486)	502.7 (444.6–559.5)	4057 (3554–4548)	559.6 (492.7–621.9)	11.3 (7.4 to 14.5)
Haiti	16,740 (14,742–18,656)	442.4 (391.7–492.7)	44,247 (39,083–49,489)	482.7 (427.1–537.4)	9.1 (5.4 to 12.8)
Jamaica	8488 (7542–9432)	512.6 (452.9–570.6)	17,247 (15,256–19,199)	560.8 (496–622.8)	9.4 (6.4 to 13)
Puerto Rico	20,664 (18,419–23,019)	579.8 (514.6–646.6)	32,519 (29,015–35,959)	625.5 (556.8–693.5)	7.9 (4.6 to 11.2)
Saint Kitts and Nevis	169 (150–187)	542.2 (477.3–601.1)	468 (410–523)	588.2 (521.2–651.6)	8.5 (5.4 to 11.5)
Saint Lucia	444 (395–492)	508 (448.1–566.4)	1385 (1221–1539)	568 (502.9–629)	11.8 (8.1 to 15.7)
Saint Vincent and the Grenadines	348 (312–388)	504.6 (449.6–563)	798 (705–893)	558.3 (492.9–624.1)	10.6 (7.4 14)
Suriname	1532 (1338–1706)	539.1 (474.7–599.4)	3896 (3417–4354)	583.1 (515.6–644.2)	8.2 (5 to 11.4)
Trinidad and Tobago	4880 (4312–5458)	542.8 (477.8–606.7)	10,980 (9707–12,243)	584.4 (517.3–650.6)	7.7 (4.8 to 11.1)
United States Virgin Islands	580 (510–648)	569.2 (503.1–631.1)	889 (784–995)	612.2 (539.7–680.7)	7.6 (4.2 to 10.7)
Central Latin America	509,648 (450,946–568,026)	528 (468.1–587.1)	1,561,605 (1,379,181–1,731,311)	589.5 (521.5–652.5)	11.7 (10.4 to 13)
Colombia	104,721 (93,002–117,051)	506.1 (447.9–565)	311,333 (273,697–346,614)	565.5 (498–628.2)	11.7 (8.2 to 15.3)
Costa Rica	9941 (8816–11,032)	515.1 (456.4–573.8)	31,517 (27,881–35,173)	573 (507.2–639.6)	11.2 (8 to 14.4)
El Salvador	16,197 (14,204–17,967)	506.5 (446.9–560.4)	34,374 (30,44,7–38,032)	570.5 (505.6–631.6)	12.6 (9.2 to 16.1)
Guatemala	19,814 (17,426–21,950)	473.7 (418.4–526.4)	63,652 (56,005–70,928)	526.8 (464.9–587.7)	11.2 (7.8 to 15.2)
Honduras	11,446 (10,046–12,716)	483.2 (423.4–537)	39,418 (34,602–43,969)	534.6 (472–594.8)	10.6 (7.3 to 14.5)
Mexico	271,007 (239,140–301,174)	549.2 (485.5–609.1)	845,739 (741,870–936,364)	617 (544.5–681.4)	12.4 (10.9 to 13.9)
Nicaragua	8668 (7628–9609)	480.8 (422.7–533.7)	30,198 (26,577–33,716)	542.5 (479.6–605.3)	12.8 (9.4 to 16.9)
Panama	8020 (7082–8912)	489.4 (432.9–543.5)	24,714 (21,961–27,431)	557.5 (495.3–619.1)	13.9 (10.3 to 17)
Venezuela (Bolivarian Republic of)	59,833 (53,028–66,428)	525.7 (464.2–586.6)	180,660 (159,653–201,259)	568.6 (504.6–632)	8.2 (5.4 to 11.2)
Tropical Latin America	551,639 (485,844–614,416)	527.6 (467.4–585.3)	1,568,533 (1,385,103–1,733,206)	589.1 (521.4–650.6)	11.7 (10.4 to 12.9)
Brazil	538,758 (474,541–599,988)	527.7 (467.5–585.5)	1,533,313 (1,353,748–1,694,670)	590 (522.2–651.5)	11.8 (10.6 to 13.1)
Paraguay	12,881 (11,367–14,300)	523.2 (462.6–582)	35,221 (31,206–39,089)	552.5 (490.2–612.9)	5.6 (2.4 to 9)
North Africa and Middle East	839,234 (738,099–934,460)	424.3 (375.4–470.7)	2,730,761 (2,402,789–3,044,967)	488.3 (433.7–542.3)	15.1 (13.5 to 16.5)
Afghanistan	27,287 (23,791–30,990)	369.7 (327.4–412.5)	57,027 (49,382–64,359)	409.7 (362.3–460.3)	10.8 (6.8 to 14.3)
Algeria	58,393 (51,616–65,377)	423.2 (376–472)	209,507 (183,864–233,743)	497.5 (436.8–552.4)	17.6 (13.3 to 21.6)
Bahrain	1352 (1195–1500)	474.1 (417.3–525.6)	8147 (7061–9159)	510.4 (450.1–570.2)	7.7 (4.5 to 10.6)
Egypt	145,297 (127,232–162,962)	426.7 (376.1–479.1)	395,500 (346,709–441,377)	485.9 (428.3–542.2)	13.9 (10.2 to 17.3)
Iran (Islamic Republic of)	132,276 (116,401–147,761)	431.9 (381.9–478.3)	449,414 (395,816–499,367)	490 (433.9–541.9)	13.4 (12.2 to 14.7)
Iraq	40,791 (35,892–45,168)	448 (392.8–498.8)	149,558 (130,581–167,726)	482.2 (424.6–538)	7.6 (4.4 to 10.6)
Jordan	7852 (6902–8789)	453.2 (403.1–504.6)	51,742 (45,552–57,345)	512.4 (455–567.6)	13.1 (10.3 to 16.3)
Kuwait	4803 (4240–5357)	472.5 (421.4–526.8)	27,284 (23,711–30,553)	531 (470.4–591)	12.4 (8.6 to 16.2)
Lebanon	10,502 (9259–11,792)	435.8 (386.3–485.7)	29,681 (26,182–32,856)	508.7 (448–564.4)	16.7 (13.3 to 20.6)
Libya	10,070 (8832–11,224)	453.8 (400.4–504.5)	35,916 (31,462–40,045)	504 (443.8–558.8)	11 (7.9 to 14.3)
Morocco	68,064 (60,212–76,074)	430.1 (380.3–479)	180,998 (159,987–202,804)	473.5 (419.5–527.3)	10.1 (6.8 to 13.7)
Oman	4164 (3668–4636)	423.7 (377.2–472)	18,373 (16,230–20,499)	501.2 (444.5–559.7)	18.3 (15.1 to 21.6)
Palestine	4112 (3617–4599)	431.5 (383.2–482.9)	15,981 (14,017–17,901)	482.5 (423.9–538.8)	11.8 (8.3 to 15.9)
Qatar	1288 (1128–1449)	471.6 (416.7–524.5)	138,22 (12,031–15,489)	518.2 (460.6–577.5)	9.9 (6.6 to 13.4)
Saudi Arabia	35,182 (30,926–39,296)	438.3 (387.1–487.7)	184,906 (161,454–206,867)	519 (457.7–575)	18.4 (14.9 to 22.4)
Sudan	39,609 (34,928–44,229)	374.8 (330.9–417.3)	114,478 (100,690–127,671)	451.1 (400.9–502)	20.4 (15.5 to 24.4)
Syrian Arab Republic	25,784 (22,779–28,805)	421.3 (376.9–468)	75,626 (65,837–85,155)	482.7 (425.8–541.9)	14.6 (10.6 to 18.6)
Tunisia	23,828 (21,006–26,756)	430 (379.5–481.7)	69,721 (61,710–77,560)	492.8 (438.3–545.9)	14.6 (11 to 18.1)
Turkiye	172,035 (151,350–191,270)	432.3 (383.5–478.7)	498,940 (442,251–557,033)	504.5 (447.6–564.5)	16.7 (12.7 to 20.5)
United Arab Emirates	4549 (3970–5115)	442.6 (391–493.6)	63,708 (55,394–72,162)	492.3 (435.3–547.2)	11.2 (8 to 14.7)
Yemen	21,538 (18,961–23,990)	363.4 (320.1–407.3)	77,885 (68,747–86,681)	421.3 (371.1–470.3)	15.9 (12 to 19.7)
South Asia	299,3308 (2,640,026–3,326,476)	430.8 (382–477.6)	8,220,378 (7,241,991–9,115,916)	495 (436.6–548)	14.9 (13.5 to 16.5)
Bangladesh	223,684 (198,212–248,765)	402.6 (355.1–449.4)	690,027 (612,220–767,881)	455.7 (405.4–506.4)	13.2 (9.8 to 16.6)
Bhutan	1259 (1112–1409)	409.5 (363.5–454.7)	3181 (2820–3532)	465.7 (411.2–516.8)	13.7 (10.5 to 17.5)
India	2,474,842 (2,182,571–2,747,485)	438.6 (389.3–485.3)	6,701,764 (5,911,471–7,428,210)	505 (445.8–559.2)	15.1 (13.7 to 16.8)
Nepal	44,229 (39,303–49,167)	387.6 (345.6–431.6)	115,211 (102,658–128,002)	446.6 (398.8–496.3)	15.2 (11.6 to 19.1)
Pakistan	249,293 (218,772–280,263)	394.1 (346.6–441.4)	710,195 (618,261–798,896)	458 (402.5–510)	16.2 (13.8 to 19.2)
East Asia	4,836,774 (4,234,495–5,416,117)	487.3 (428.3–544)	12,051,700 (10,562,111–13,556,244)	554.5 (486.9–619.4)	13.8 (11.9 to 15.9)
China	4,654,141 (4,075,192–5,212,886)	487.1 (428.1–543.8)	11,652,721 (10,207,638–13,107,929)	554.6 (486.9–619.5)	13.9 (12 to 16.1)
Democratic People’s Republic of Korea	90,679 (79,003–103,017)	473 (414.9–528.3)	175,530 (153,632–197,148)	505.1 (444.9–562.6)	6.8 (2.5 to 10.5)
Taiwan	91,954 (80,996–102,954)	519.3 (456.8–584.2)	223,449 (197,854–249,986)	594.3 (526.1–663.5)	14.4 (10.7 to 18.1)
Oceania	16,659 (14,676–18,607)	441.7 (389.8–491.7)	47,743 (41,835–53,394)	481 (423.1–536.4)	8.9 (6.2 to 11.4)
American Samoa	160 (140–178)	547.3 (481.3–608.2)	323 (284–361)	592.9 (525.3–658.1)	8.3 (5.1 to 11.4)
Cook Islands	75 (66–84)	525 (466.3–583.3)	141 (124–158)	600.1 (530.1–670)	14.3 (10.5 to 17.6)
Fiji	2369 (2091–2656)	489.4 (434.7–547.8)	5075 (4459–5702)	561.8 (494.8–628)	14.8 (11 to 18.3)
Guam	525 (461–586)	526.1 (460–585.5)	1157 (1019–1289)	585.1 (516.2–653.5)	11.2 (7.8 to 14.9)
Kiribati	224 (196–249)	499.1 (440–557.5)	496 (436–556)	543 (480.8–604.8)	8.8 (5.4 to 11.8)
Marshall Islands	97 (85–107)	483.8 (425.4–539.6)	254 (222–284)	540.8 (474.6–600.3)	11.8 (8.6 to 14.9)
Micronesia (Federated States of)	267 (237–296)	489.5 (430.6–544)	500 (435–560)	550.9 (485.3–611.8)	12.5 (9.3 to 15.9)
Nauru	31 (27–34)	498.6 (439.2–555.2)	42 (37–47)	565.6 (496.8–627.8)	13.4 (9.9 to 17.8)
Niue	11 (9–12)	520.5 (460.9–581.9)	12 (11–14)	584.1 (517.3–647.9)	12.2 (8.6 to 16.1)
Northern Mariana Islands	165 (145–185)	525.9 (465.5–584.3)	362 (314–410)	571.4 (503.6–639.8)	8.7 (5.1 to 12.2)
Palau	59 (52–66)	523.5 (461.2–583.2)	156 (136–176)	577.3 (510.6–644)	10.3 (7 to 14)
Papua New Guinea	9687 (8501–10,874)	418.2 (367.9–466.1)	32,236 (28,233–36,150)	458.5 (402.6–512.1)	9.6 (5.7 to 13.3)
Samoa	477 (419–533)	501.8 (442.9–559)	906 (796–1019)	554.5 (488.2–621.2)	10.5 (7 to 14)
Solomon Islands	762 (666–855)	444.6 (392.6–495.5)	2344 (2039–2634)	506.2 (444.4–563.7)	13.8 (10.1 to 17.6)
Tokelau	6 (5–7)	485.3 (428–541.9)	8 (7–9)	561.1 (496–621.9)	15.6 (11.6 to 19.8)
Tonga	303 (265–338)	493.7 (435.2–550)	469 (412–524)	548.8 (483.1–609.6)	11.1 (7.5 to 15.7)
Tuvalu	36 (32–40)	483.2 (425.5–536.4)	61 (53–68)	547 (481.1–611.5)	13.2 (9.8 to 16.8)
Vanuatu	343 (299–381)	429.5 (376.8–474.4)	1056 (931–1190)	479.6 (425.5–537.9)	11.7 (8.2 to 15.1)
Southeast Asia	1,128,950 (993,293–1,259,580)	376 (332.3–418.4)	3,261,525 (2,872,405–3,641,133)	437.1 (386.1–485)	16.2 (14.5 to 18.1)
Cambodia	18,793 (16,448–21,070)	349.2 (307.3–389.7)	56,903 (50,231–63,538)	395.3 (350.2–440.7)	13.2 (9.3 to 17.9)
Indonesia	457,055 (400,852–509,726)	383.1 (337.8–426.1)	1,308,806 (1,146,853–1,469,965)	446.3 (394–496.7)	16.5 (14.6 to 18.5)
Lao People’s Democratic Republic	8442 (7403–9418)	353.4 (312–393.8)	23,167 (20,272–25,801)	402.5 (353.2–448.2)	13.9 (10 to 18.6)
Malaysia	46,814 (41,144–52,105)	411.6 (363–457.7)	149,066 (130,992–165,937)	473.7 (417.8–527)	15.1 (11.5 to 18.8)
Maldives	443 (384–497)	394.8 (347.2–439.8)	2285 (1997–2542)	456.2 (400.2–506.7)	15.5 (11.8 to 19.1)
Mauritius	3554 (3137–3952)	426.2 (374.8–474.8)	8641 (7560–9666)	483.3 (426.4–540.1)	13.4 (10 to 16.5)
Myanmar	94,100 (83,053–104,576)	358.7 (318–397.3)	233,234 (205,139–260,511)	423.5 (373.2–470)	18 (13.5 to 22.3)
Philippines	136,805 (119,815–152,614)	379.1 (333.4–423.8)	419,469 (366,178–471,536)	436.1 (382.6–486)	15 (13.9 to 16.3)
Seychelles	232 (206–258)	425.8 (375.9–474.2)	635 (552–713)	484.3 (424.3–539)	13.7 (10 to 17.7)
Sri Lanka	48,932 (42,912–54,503)	380.9 (336.2–423.9)	117,977 (103,435–131,691)	438.2 (385.5–486.7)	15 (11 to 19)
Thailand	165,162 (144,619–183,600)	383.5 (337.9–423.8)	484,228 (425,932–544,645)	462.7 (408.9–515.7)	20.7 (16 to 25.7)
Timor-Leste	1405 (1232–1580)	348.4 (306.3–389.2)	3682 (3244–4098)	400.4 (353–446)	14.9 (11 to 18.8)
Viet Nam	145,579 (128,053–162,688)	351.5 (310.2–392.7)	448,882 (391,923–502,050)	400.8 (350.7–445.4)	14 (9.6 to 18.2)
Central Sub-Saharan Africa	122,007 (106,783–135,467)	441.9 (390.4–491.2)	339,701 (298,431–378,043)	463.1 (409.7–515.2)	4.8 (2.4 to 7.2)
Angola	24,001 (21,033–26,933)	467.3 (411.2–518.9)	83,357 (72,517–93,086)	512.7 (451.4–570.5)	9.7 (6.5 to 13.1)
Central African Republic	5884 (5145–6581)	414.9 (365.6–461.8)	13,042 (11,395–14,583)	429.1 (378.8–478.1)	3.4 (0 to 6.4)
Congo	5796 (5101–6479)	465.4 (408.9–518.5)	19,069 (16,720–21,209)	507 (449.6–563.6)	8.9 (5.6 to 12.1)
Democratic Republic of the Congo	82,694 (72,458–92,054)	435.4 (383.4–484)	213,657 (186,934–237,906)	443 (392.6–492.3)	1.7 (-1.6 to 4.9)
Equatorial Guinea	929 (813–1044)	404.6 (357.5–451.5)	3776 (3311–4195)	525.1 (463.7–583.6)	29.8 (24.7 to 34.9)
Gabon	2703 (2373–3027)	449.4 (394.8–501.7)	6801 (5969–7588)	521.5 (462.1–581.1)	16 (12.7 to 19.9)
Eastern Sub-Saharan Africa	366,643 (322,665–409,382)	411.5 (363.2–457.5)	1,000,971 (885,084–1,115,451)	461 (407.4–509.9)	12 (10.4 to 13.5)
Burundi	10,533 (9293–11,674)	399.8 (353.2–444.9)	26,112 (22,940–29,320)	408.8 (361.2–455.1)	2.2 (-0.2 to 4.9)
Comoros	956 (838–1072)	410.2 (362–460)	2636 (2323–2942)	453.3 (401–504.2)	10.5 (7.1 to 13.8)
Djibouti	781 (687–876)	406.3 (358.1–454)	4368 (3830–4867)	475.2 (421.5–528.2)	16.9 (13.1 to 21)
Eritrea	6020 (5275–6752)	387.2 (342.8–433.2)	16,113 (14,179–18,023)	424.4 (373.7–470.4)	9.6 (6 to 13.7)
Ethiopia	99,638 (87,385–111,462)	416.4 (368.1–462.5)	270,742 (237,203–300,718)	497.7 (438.5–554.7)	19.5 (15.6 to 23)
Kenya	43,389 (38,340–48,283)	447.6 (396–495.1)	151,394 (132,775–168,327)	516.3 (457.2–572.7)	15.4 (13.6 to 17)
Madagascar	22,701 (19,851–25,438)	387.8 (341.6–435.9)	62,380 (54,683–69,430)	408.2 (360.9–454.3)	5.3 (2.1 to 8.6)
Malawi	18,372 (16,139–20,422)	401.6 (356.1–448.5)	41,599 (36,587–46,466)	441.2 (389.3–493.5)	9.8 (6.5 to 13.6)
Mozambique	28,521 (25,087–31,792)	393.9 (348–440)	62,053 (54,601–69,191)	432.7 (382.5–481.3)	9.8 (6.1 to 13.5)
Rwanda	13,013 (11,507–14,540)	393.2 (349.2–439.8)	33,948 (29,699–37,791)	426.6 (375.2–474.4)	8.5 (4.8 to 12.3)
Somalia	13,591 (11,944–15,234)	397.6 (350.1–443)	35,857 (31,435–40,006)	413.5 (365.1–463.1)	4 (0.5 to 7.1)
South Sudan	11,184 (9858–12,479)	391.7 (345.9–437)	22,010 (19,217–24,633)	419.3 (370.7–468.5)	7.1 (3.7 to 11)
Uganda	29,418 (25,828–33,000)	393.4 (345.2–440.7)	82,029 (72,113–91,566)	432.9 (383.3–481.7)	10 (6.4 to 13.6)
United Republic of Tanzania	53,272 (46,769–59,337)	425.3 (376.5–472.6)	145,867 (129,292–162,102)	453.7 (402.9–505)	6.7 (3.5 to 10)
Zambia	14,992 (13,119–16,795)	430.5 (379.5–480.1)	42,993 (37,783–48,108)	453.8 (400.9–505)	5.4 (2.3 to 9.1)
Southern Sub-Saharan Africa	157,577 (139,016–175,112)	513.3 (454.8–568.8)	375,228 (330,227–417,302)	557.2 (493.5–618.2)	8.6 (7.3 to 9.7)
Botswana	3012 (2675–3379)	456.8 (405.5–509.5)	10,298 (9103–11,420)	535.6 (473.8–595.8)	17.3 (13.4 to 21)
Eswatini	1688 (1488–1891)	468.6 (414.6–521)	3821 (3367–4244)	533.1 (470.1–591.8)	13.8 (9.4 to 17.5)
Lesotho	4036 (3571–4498)	433.5 (383.8–484.2)	6345 (5633–7046)	504.3 (447.1–559.2)	16.3 (12.1–20.2)
Namibia	3250 (2855–3639)	435.7 (384.5–486.9)	8344 (7334–9310)	491.3 (434.6–546.4)	12.8 (9 to 16.3)
South Africa	125,033 (110,150–139,040)	538.5 (477–598.3)	306,662 (269,896–340,955)	579.7 (513.4–643.7)	7.6 (6.3 to 9)
Zimbabwe	20,557 (18,221–22,842)	429.3 (381.9–477.1)	39,758 (34,864–44,366)	447.9 (394.3–502.7)	4.3 (0.9 to 7.3)
Western Sub-Saharan Africa	448,326 (395,308–501,301)	439.6 (387.3–489.1)	1,226,622 (1,078,017–1,368,997)	483.8 (427.1–536.7)	10.1 (9.1 to 11)
Benin	9508 (8400–10,546)	427.3 (379.1–475.6)	32,569 (28,507–36,258)	492.7 (436–545.2)	15.3 (11.5 to 19)
Burkina Faso	19,593 (17,143–21,971)	399.7 (353.5–447.9)	49,304 (43,079–54,826)	434.6 (382.8–485.6)	8.7 (5.1 to 12.4)
Cabo Verde	878 (774–988)	432 (381.5–486)	2533 (2238–2845)	499.3 (442.5–558.2)	15.6 (11.6 to 19.4)
Cameroon	23,980 (21,001–26,765)	448.3 (396.4–501.4)	82,772 (73,056–92,140)	498 (442.5–554.9)	11.1 (7.6 to 14.5)
Chad	12,181 (10,745–13,631)	399.8 (352.6–447.5)	30,987 (27,232–34,602)	420.7 (371.5–469.7)	5.2 (1.6 to 8.4)
Côte d’Ivoire	23,224 (20,352–25,968)	430.1 (380–479.3)	73,190 (63,804–81,621)	473.8 (419.3–528.2)	10.1 (7 to 13.6)
Gambia	1849 (1624–2066)	426.6 (376.6–478.6)	6003 (5267–6646)	491.2 (432.8–544.3)	15.1 (11.4 to 18.8)
Ghana	37,545 (32,916–41,932)	483.2 (426.3–536.3)	110,976 (96,895–123,575)	514.7 (452.4–573.1)	6.5 (3.2 to 10.3)
Guinea	14,609 (12,925–16,366)	407.5 (360.2–458.2)	29,814 (26,205–33,040)	441.3 (391–490.6)	8.3 (4.8 to 12.1)
Guinea-Bissau	1959 (1719–2194)	415.9 (366.2–467)	4439 (3883–4937)	449.9 (396.6–504.3)	8.2 (4.4 to 11.5)
Liberia	5476 (4876–6106)	432.3 (384.1–485.4)	14,830 (13,025–16,592)	483.8 (427.6–539.8)	11.9 (8.7 to 15.3)
Mali	18,970 (16,612–21,294)	404.8 (356.4–453.4)	48,917 (42,975–54,786)	445 (394.4–496.9)	9.9 (6.3 to 13.2)
Mauritania	4903 (4315–5483)	443 (390.8–495.6)	12,721 (11,194–14,181)	500.5 (441.3–558.3)	13 (9.9 to 16.6)
Niger	14,144 (12,459–15,854)	399.9 (353.2–443.5)	43,202 (38,133–48,221)	423.8 (375.5–472.8)	6 (2.7 to 9.4)
Nigeria	227,291 (200,074–254,306)	450.2 (398–500.7)	593,768 (521,287–662,832)	498.7 (440.8–553.2)	10.8 (9.5 to 12.1)
Sao Tome and Principe	299 (264–335)	449.8 (398.3–501.9)	764 (669–851)	524.2 (462.2–582.2)	16.6 (12.7 to 20.3)
Senegal	16,080 (14,163–17,955)	433.8 (381.8–484)	44,161 (38,546–49,139)	475.5 (416.8–528.9)	9.6 (6 to 13.2)
Sierra Leone	9247 (8198–10,287)	410.1 (361.8–459.5)	21,582 (18,976–23,937)	456.7 (405–507.8)	11.4 (7.3 to 15.3)
Togo	6576 (5783–7297)	421.5 (372.3–469.4)	24,075 (21,234–26,922)	469.2 (415.2–524.3)	11.3 (7.6 to 15.7)

UI = uncertainty intervals.

**Figure 3.**
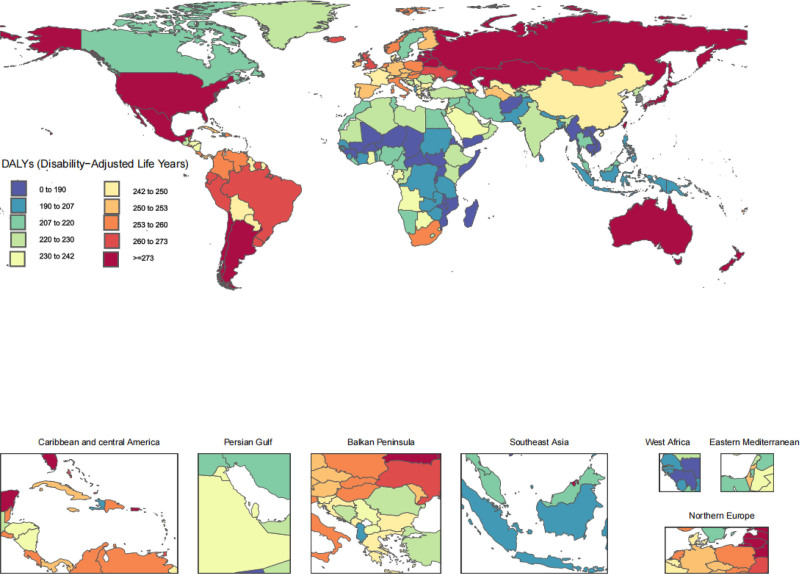
Age-standardized DALYs rates estimates of osteoarthritis in 2021 at national level Age and sex patterns. DALYs = disability-adjusted life years.

In 2021, the Republic of Korea, Singapore, Brunei Darussalam, and the United States of America had the highest age-standardized DALYs. Specifically, the Republic of Korea had a rate of 327.1 (with a 95% UI of 157.4–662.8), Singapore had a rate of 323.8 (95% UI 156.1–48.8), Brunei Darussalam had a rate of 319.0 (95% UI 152.8–642.4), and the United States of America had a rate of 310.8 (95% UI 149.7–627.3). In contrast, Afghanistan, Madagascar, Cambodia, and South Sudan had the lowest age-standardized DALYs in 2021. Afghanistan had a rate of 168.7 (95% UI 82.6–342.5), Madagascar had a rate of 172.4 (95% UI 82.8–346.8), Cambodia had a rate of 172.5 (95% UI 82.6–347.1), and South Sudan had a rate of 173.7 (95% UI 83.9–349.8). Between 1990 and 2021, the most significant increases in age-standardized DALYs were observed in Equatorial Guinea, which saw a rise of 40.2% (95% UI 33.7–46.7%), followed by Mongolia with an increase of 30.1% (95% UI 24.6–36.2%), Ethiopia, where the rate went up by 26.9% (95% UI 21.6–31.5%), and Thailand, which experienced an increase of 24.7% (95% UI 19.5–30%). In contrast, Denmark was the only country to exhibit a slight downward trend, with a decrease of 3.6% (95% UI −8.7% to 2.0%) (Table 4). Figure 3 shows the distribution of DALYs due to osteoarthritis across countries in 2021, with darker colors indicating higher DALYs values.

**Table 4:** DALYs due to osteoarthritis in 1990 and 2021 and the percentage change in the age-standardised rates (ASRs) per 100,000, by location (generated from data available from http://ghdx.healthdata.org/gbd-results-tool).

Location	1990		2021		Percentage change in the ASRs per 100,000
	No. (95% UI)	ASRs per 100,000 (95% UI)	No (95% UI)	ASRs per 100,000 (95% UI)	
Global	8,918,857 (4,264,151–1,7983,776)	222.8 (106.6–450.3)	21,304,566 (10,189,161–42,935,420)	244.5 (117.1–493.1)	9.7 (9.1 to 10.4)
Central Asia	101,371 (48,802–204,167)	215.8 (104.2–434.3)	212,365 (101,660–425,622)	249.3 (119.6–500.6)	15.5 (13.1 to 17.9)
Armenia	5467 (2629–10,900)	196.8 (94.9–394.9)	10,518 (5107–21,122)	243.5 (117.9–489.8)	23.7 (19 to 29.4)
Azerbaijan	11,420 (5454–23,078)	226 (108.2–455.6)	27,348 (13,227–54,529)	250.2 (121.3–498.4)	10.7 (6.5 to 15)
Georgia	13,507 (6569–27,037)	214.4 (104–429.7)	13,536 (6551–27,155)	231.7 (111.8–465.1)	8.1 (4.4 to 12)
Kazakhstan	29,186 (13,995–58,957)	229.7 (110.4–463.6)	51,050 (24,479–103,161)	275.7 (132.5–558)	20.1 (15.6 to 24.5)
Kyrgyzstan	6397 (3090–12,804)	215.4 (103.8–431.7)	12,276 (5873–24,609)	243.6 (116.4–488.6)	13.1 (9.1 to 17.4)
Mongolia	2095 (1001–4190)	200.5 (96.1–401.5)	6453 (3067–13,169)	260.9 (124.7–529.1)	30.1 (24.6 to 36.2)
Tajikistan	5145 (2472–10,395)	188.2 (90.4–379.3)	12,933 (6153–25,853)	207.4 (99.1–415.7)	10.2 (6.4 to 14.5)
Turkmenistan	3946 (1889–7988)	207.1 (99.3–418.5)	10,538 (4991–20,938)	250.9 (118.9–501.5)	21.1 (16.4 to 26.5)
Uzbekistan	24,208 (11,686–48,890)	211.1 (102.1–426.4)	67,714 (32,354–135,489)	244.6 (116.9–490.4)	15.9 (12.1 to 20)
Central Europe	327,001 (157,196–659,825)	218.6 (104.7–441.8)	514,447 (248,117–1,044,284)	245.4 (117.6–496.2)	12.2 (10.9 to 13.6)
Albania	3649 (1770–7291)	172.7 (84.2–345.2)	8677 (4127–17,605)	202.5 (95.8–411.3)	17.2 (12.8 to 21.7)
Bosnia and Herzegovina	8100 (3852–16,233)	190.8 (90.9–382.3)	13,707 (6543–27,707)	228.1 (108.2–460.6)	19.5 (15.2 to 24.1)
Bulgaria	27,784 (13,267–55,752)	223.8 (106.2–449.8)	33,207 (16,137–67,206)	247.9 (119.9–500.1)	10.8 (7.2 to 14.5)
Croatia	13,850 (6614–28,014)	222.5 (105.9–451.2)	19,991 (9654–40,472)	242.8 (116.2–490.5)	9.1 (5.4 to 12.7)
Czechia	31,731 (15,388–64,337)	234.9 (113.6–476.2)	50,207 (24,413–101,811)	252.4 (122.4–511.4)	7.4 (4.1 to 10.8)
Hungary	33,900 (16,394–68,586)	233.8 (112.6–473.4)	46,542 (22,390–93,906)	255.4 (122–513.4)	9.2 (5.8 to 12.2)
Montenegro	1460 (700–2946)	230.3 (110.5–464.4)	2383 (1146–4868)	246.5 (117.9–504.2)	7.1 (3.3 to 10.3)
North Macedonia	3957 (1891–8023)	206.5 (98.9–420)	7720 (3730–15,667)	230.9 (111.1–468.2)	11.8 (8.1 to 15.2)
Poland	96,022 (46,325–193,634)	220.8 (106.1–445.9)	173,974 (84,081–351,958)	255.4 (122.7–515)	15.7 (14 to 17.5)
Romania	57,922 (27,934–116,376)	205.8 (98.8–413.9)	80,580 (38,213–163,255)	229.1 (108.1–463.2)	11.3 (7.9 to 14.9)
Serbia	24,007 (11,469–48,135)	207.2 (98.3–417.6)	36,404 (17,445–73,708)	233.4 (111.1–471.9)	12.7 (8.6 to 16.5)
Slovakia	13,706 (6488–27,895)	231.9 (109.6–472)	23,395 (11,270–47,393)	253.8 (121.5–514.8)	9.4 (6.2 to 13.3)
Slovenia	5683 (2695–11,431)	231.2 (109.6–465.7)	10,173 (4918–20,701)	249.7 (119.9–506.2)	8 (4.4 to 11.5)
Eastern Europe	742,363 (355,456–1,508,177)	265.8 (126.8–540.9)	965,580 (465,099–1,951,745)	280.8 (134.4–567)	5.6 (4.2 to 6.9)
Belarus	31,504 (15,063–64,043)	243 (115.9–494.7)	43,468 (20,750–86,960)	276.6 (131–552.9)	13.8 (10 to 17.5)
Estonia	5205 (2498–10,404)	255.2 (122.1–509.7)	7010 (3368–14,040)	288.8 (137.7–578.2)	13.2 (9.4 to 16.7)
Latvia	8834 (4205–17,934)	247.5 (117.6–502.4)	10,205 (4911–20,814)	282.1 (134.9–576.8)	14 (10.5 to 18)
Lithuania	10,970 (5260–22,184)	243.2 (116.4–491.9)	14,730 (7078–30,377)	278.3 (132.6–574.6)	14.4 (11 to 18.6)
Republic of Moldova	9527 (4560–19,321)	215 (102.7–436.5)	14,658 (7021–29,353)	250.3 (119.7–501.9)	16.4 (12.6 to 20.6)
Russian Federation	502,937 (239,784–1,018,933)	278.8 (132.6–565.8)	673,208 (324,712–1,363,422)	286.2 (137.1–579.3)	2.7 (1.1 to 4.3)
Ukraine	17,3385 (84,207–350,222)	243.2 (118–492.1)	202,302 (97,312–406,820)	267.2 (127.6–536.3)	9.9 (6.5 to 13.4)
Australasia	58,807 (28,504–118,543)	254.5 (123.2–513.8)	140,906 (69,472–286,856)	283.4 (139.2–578)	11.4 (8 to 14.6)
Australia	49,048 (23,740–98,909)	254.5 (122.9–514)	118,306 (58,626–241,249)	283.7 (140–579.9)	11.5 (7.6 to 15.3)
New Zealand	9759 (4730–19,669)	254.1 (122.9–512.4)	22,600 (10,846–45,607)	281.6 (134.6–568.2)	10.8 (7.8 to 14.1)
High-income Asia Pacific	597,447 (286,964–1,207,310)	291.1 (139.9–588)	1,276,815 (611,789–2,579,654)	315 (150.5–636.8)	8.2 (6.7 to 9.8)
Brunei Darussalam	322 (153–649)	295.7 (141.5–598.4)	1223 (583–2448)	319 (152.8–642.4)	7.9 (4.7 to 11.2)
Japan	493,608 (237,043–994,224)	287 (137.9–578.1)	939,485 (450,469–1,907,692)	309.5 (147.6–625.2)	7.8 (6.3 to 9.5)
Republic of Korea	96,296 (46,363–195,130)	310.3 (149.9–629.4)	307,677 (148,215–623,772)	327.1 (157.4–662.8)	5.4 (2.1 to 8.5)
Singapore	7222 (3412–14,318)	312.3 (148.9–621.2)	28,430 (13,694–56,974)	323.8 (156.1–648.8)	3.7 (0.7 to 7)
High-income North America	964,587 (463,191–1,948,963)	286.2 (137.2–576.1)	1,857,796 (900,403–3,761,748)	300.9 (144.9–607)	5.1 (4.1 to 6.3)
Canada	63,958 (31,072–130,703)	200.6 (97.2–409.5)	146,845 (71,051–296,532)	219.1 (105.4–442)	9.2 (5.6 to 12.7)
Greenland	72 (35–148)	199.7 (96.7–407.6)	162 (78–327)	221.7 (106.6–445.9)	11 (7.7 to 14.7)
United States of America	900,534 (432,395–1,819,200)	295.3 (141.5–594.2)	1,710,760 (829,259–3,463,603)	310.8 (149.7–627.3)	5.2 (4.2 to 6.4)
Southern Latin America	115,112 (54,896–232,022)	248 (118.3–500)	233,580 (112,227–468,574)	273.5 (131.2–548.7)	10.3 (7.7 to 12.9)
Argentina	80,960 (38,452–162,325)	249.8 (118.7–501.1)	150,107 (71,902–303,302)	273.8 (131.2–552.1)	9.6 (6 to 12.9)
Chile	24,662 (11,880–50,151)	242 (116.9–492.1)	69,421 (33,532–138,326)	273 (132.2–543.9)	12.8 (9.3 to 16.5)
Uruguay	9485 (4532–18,885)	248.3 (118.5–493.4)	14,039 (6795–27,922)	273 (131.6–540.8)	9.9 (6.4 to 13.6)
Western Europe	1,327,348 (642,543–2,667,248)	238.6 (115.2–479.9)	2,131,317 (1,038,440–4,287,323)	253.6 (123.1–510.6)	6.3 (5.2 to 7.5)
Andorra	136 (66–277)	234 (112.7–475.8)	384 (184–765)	251.1 (120.5–499.6)	7.3 (4.5 to 10.9)
Austria	26,567 (12,792–53,034)	235.4 (112.9–471.6)	41,716 (20,003–84,034)	250.2 (119.6–502.8)	6.3 (3 to 9.9)
Belgium	34,844 (16,683–69,417)	235.9 (112.6–469.9)	52,625 (25,528–106,465)	249.1 (120.1–503.7)	5.6 (2.4 to 9.1)
Cyprus	1818 (873–3643)	220.5 (106–442.2)	4956 (2403–9910)	244.8 (118.5–489.7)	11 (7.9 to 14.9)
Denmark	19,538 (9558–39,976)	256.2 (125.2–525.2)	26,730 (12,923–54,646)	246.8 (119.1–502.5)	-3.6 (-8.7 to 2)
Finland	16,277 (7928–32,884)	234.4 (113.9–474.6)	27,939 (13,503–56,190)	250.4 (120.1–504.9)	6.8 (3.7 to 10.2)
France	185,036 (89,540–372,835)	234.4 (113–472.9)	310,428 (150,027–625,921)	249.8 (120–501.6)	6.6 (3.2 to 10)
Germany	294,049 (142,785–593,358)	240.3 (116.6–486.3)	437,791 (215,923–879,846)	252.4 (124.2–508.2)	5 (1.3 to 8.3)
Greece	32,944 (15,987–66,127)	220.3 (106.7–442.5)	51,134 (24,852–103,742)	242.7 (117.5–488.9)	10.2 (6.6 to 13.8)
Iceland	707 (346–1452)	257.1 (125.6–529.6)	1447 (705–2897)	266.6 (129.2–534.8)	3.7 (-1.4 to 8.4)
Ireland	9217 (4440–18,588)	232.1 (111.8–469.5)	19,052 (9230–38,297)	251 (121.5–505.3)	8.1 (4.8 to 11.4)
Israel	11,093 (5293–22,315)	234.4 (111.8–470.3)	29,412 (14,050–59,275)	251.5 (119.8–507.2)	7.3 (4.3 to 10.5)
Italy	206,445 (99,872–413,614)	238.9 (115.3–479.4)	328,910 (159,768–663,711)	253.8 (122.4–511.5)	6.2 (5.1 to 7.3)
Luxembourg	1277 (620–2581)	238.8 (115.5–483.4)	2576 (1256–5200)	251.9 (122.5–509.1)	5.5 (2.1 to 8.5)
Malta	1011 (492–2020)	236.8 (115.3–472.4)	2227 (1083–4473)	252.9 (122.6–508.2)	6.8 (3.2 to 10.3)
Monaco	157 (75–320)	248.8 (118.9–504)	225 (109–451)	259.8 (124.7–520.5)	4.4 (0.9 to 8.1)
Netherlands	48,436 (23,580–97,161)	250.7 (121.9–504.4)	83,317 (40,222–169,267)	256.3 (123.4–520.4)	2.3 (-2.9 to 7.4)
Norway	15,181 (7350–30,580)	241.2 (116.2–483.9)	23,939 (11,585–48,115)	257.9 (124.4–518.6)	6.9 (5.4 to 8.2)
Portugal	30,963 (14,989–62,443)	225.4 (109.1–455.7)	54,607 (26,327–109,973)	247.3 (118.8–498.3)	9.7 (6.4 to 13.3)
San Marino	81 (40–163)	241.4 (118–484.7)	171 (83–345)	255.3 (123.3–515.4)	5.8 (2.8 to 8.8)
Spain	123,888 (59,793–248,852)	233.1 (112.1–466.7)	224,792 (108,763–454,456)	252.4 (121.5–509.8)	8.3 (4.6 to 12)
Sweden	28,114 (13,223–57,854)	201 (94.5–412.8)	42,255 (20,046–86,461)	219.9 (103.6–446.3)	9.4 (6.1 to 13.2)
Switzerland	23,208 (11,274–46,798)	234.9 (114–474)	40,162 (19,425–80,500)	243.3 (117–483.6)	3.6 (0.8 to 6.8)
United Kingdom	215,273 (104,015–432,201)	252 (121.3–505.5)	322,643 (156,642–645,022)	271 (130.9–542.7)	7.6 (6.8 to 8.4)
Andean Latin America	48,494 (23,257–97,370)	231.9 (111.2–466.2)	156,693 (75,061–316,245)	260.9 (125.2–526.8)	12.5 (10.2 to 15)
Bolivia (Plurinational State of)	7050 (3371–14,208)	214.7 (103.1–434.1)	23,289 (11,170–47,120)	246.7 (118.9–499.9)	14.9 (10.8 to 18.8)
Ecuador	12,890 (6151–25,627)	238.2 (113.5–474)	44,084 (21,040–87,905)	266 (127.2–531.3)	11.7 (8.1 to 15.2)
Peru	28,554 (13,791–57,638)	233.7 (113–473)	89,320 (42,787–180,731)	262.4 (125.9–531.7)	12.3 (8.7 to 15.8)
Caribbean	59,541 (28,499–120,118)	228.5 (109.5–461.2)	135,591 (64,908–274,656)	251.1 (120.1–508.3)	9.9 (8 to 11.8)
Antigua and Barbuda	125 (59–252)	245.1 (115.8–494.5)	291 (139–586)	261.9 (125.1–527)	6.8 (3.7 to 10.7)
Bahamas	403 (191–810)	256.3 (122.5–515.7)	1159 (558–2332)	270.5 (130.5–543.6)	5.5 (2.1 to 9.1)
Barbados	688 (322–1389)	253 (118.7–511.8)	1366 (650–2779)	271 (128.6–550.7)	7.1 (3.8 to 10.6)
Belize	210 (101–419)	225.9 (109.2–451.9)	817 (391–1664)	259.8 (124.6–532)	15 (11.6 to 19.2)
Bermuda	170 (81–345)	269.5 (127.8–547.6)	359 (173–735)	280.8 (134–577.7)	4.2 (0.9 to 7.6)
Cuba	23,142 (11,051–46,780)	226.6 (108.2–458.5)	48,464 (23,029–97,797)	252.1 (119.7–508.8)	11.2 (7.3 to 14.8)
Dominica	132 (63–267)	228.9 (109.8–461.7)	214 (103–429)	251.7 (120.6–504.8)	10 (6.3 to 13.4)
Dominican Republic	8702 (4128–17,380)	229.6 (108.9–460.3)	25,997 (12,460–52,426)	256.7 (123.4–517.3)	11.8 (8.2 to 15.5)
Grenada	153 (74–309)	225.2 (108–454.1)	297 (142–601)	252 (120–509)	11.9 (8.5 to 16)
Guyana	849 (407–1720)	218.6 (105.3–444.7)	1660 (797–3373)	247.4 (118.9–502.6)	13.2 (9 to 16.9)
Haiti	6140 (2993–12,364)	185.9 (90.8–375.7)	15,722 (7588–31,427)	205.6 (99.6–414.7)	10.6 (6.3 to 15)
Jamaica	3938 (1875–7953)	228.4 (108.9–461.4)	7805 (3739–15,767)	251.5 (120.3–507.7)	10.1 (6.7 to 14)
Puerto Rico	9505 (4542–19,067)	263.8 (126.2–528.3)	18,333 (8816–37,053)	285.7 (136.9–575.2)	8.3 (4.5 to 11.7)
Saint Kitts and Nevis	86 (41–173)	244 (116.8–490.2)	199 (94–401)	267.4 (127.2–537.2)	9.6 (6.2 to 13.2)
Saint Lucia	193 (93–392)	222.6 (107.6–452.8)	623 (300–1258)	254.2 (122.6–513.3)	14.2 (10.1 to 18.3)
Saint Vincent and the Grenadines	156 (74–314)	223.2 (106.2–449.8)	361 (175–744)	248.5 (120.1–511.4)	11.4 (7.7 to 15.1)
Suriname	640 (304–1304)	243.8 (115.7–497.4)	1719 (826–3439)	263.4 (126.6–526.3)	8.1 (4.5 to 11.9)
Trinidad and Tobago	2063 (981–4167)	244 (116.2–492.3)	5133 (2478–10,560)	263.6 (127.1–542.3)	8 (4.7 to 11.8)
United States Virgin Islands	231 (110–464)	258.5 (124.5–520.1)	483 (234–963)	278.1 (133.6–553.8)	7.6 (4.3 to 11.1)
Central Latin America	197,933 (94,851–39,9110)	231.7 (110.8–468)	67,6745 (322,649–1,368,410)	264.6 (126.1–535.6)	14.2 (12.8 to 15.8)
Colombia	40,680 (19,450–81,303)	223.9 (106.6–450)	142,073 (67,444–289,100)	255.9 (121.4–520.4)	14.3 (10.6 to 18.1)
Costa Rica	4032 (1924–8121)	228.1 (109–460)	14,232 (6860–28,750)	257.2 (124–519.2)	12.8 (9.4 to 16.1)
El Salvador	6668 (3152–13,494)	222.8 (105.1–451)	15,548 (7439–31,269)	254.7 (121.8–512.2)	14.3 (10.5 to 18.5)
Guatemala	7375 (3516–14,878)	204.2 (97.4–411)	25,806 (12,422–52,785)	229.3 (110.6–468.9)	12.3 (8.5 to 16.3)
Honduras	4416 (2127–8787)	210.7 (101.7–419.6)	15,483 (7463–31,057)	234.5 (113.6–471.5)	11.3 (7.9 to 15.3)
Mexico	104,803 (50,162–212,108)	239.5 (114.5–484.8)	360,985 (172,241–727,106)	277.1 (132.5–558.9)	15.7 (13.9 to 17.6)
Nicaragua	3277 (1583–6602)	207.9 (100.1–419.3)	12,087 (5854–24,322)	238.9 (116–483)	14.9 (11.2 to 19)
Panama	3269 (1562–6570)	216.4 (103.1–435.9)	11,103 (5341–22,255)	250.6 (120.4–502.6)	15.8 (11.8 to 19.4)
Venezuela (Bolivarian Republic of)	23,414 (11,253–46,677)	235.3 (113.4–469.7)	79,429 (38,221–160,697)	256.8 (123.6–519.6)	9.1 (6 to 12.8)
Tropical Latin America	213,399 (102,015–428,853)	228.1 (109.1–460.1)	678,602 (325,615–1,368,810)	259.9 (124.7–524.6)	13.9 (12.6 to 15.4)
Brazil	208,124 (99,503–418,168)	228 (109–459.8)	663,902 (318,774–1,339,005)	260.2 (125–525.2)	14.1 (12.7 to 15.7)
Paraguay	5276 (2520–10,630)	234.4 (112.3–472.6)	14,700 (6968–29,805)	246.4 (116.8–501)	5.1 (1.6 to 8.5)
North Africa and Middle East	319,580 (153,014–643,843)	183.4 (87.8–371.8)	1,049,857 (503,111–2,115,549)	215.9 (103.4–437.6)	17.7 (15.9 to 19.6)
Afghanistan	10,778 (5227–21,149)	151.9 (74.2–298.6)	18,085 (8775–36,669)	168.7 (82.6–342.5)	11 (7 to 15.5)
Algeria	23,230 (11,038–47,247)	183.4 (87.5–373.2)	84,150 (40,665–169,769)	222 (107.8–449.7)	21 (16.7 to 25.4)
Bahrain	439 (206–900)	216.4 (103–444.9)	2766 (1306–5572)	232.1 (111.3–470.1)	7.3 (4 to 10.8)
Egypt	52,977 (25,225–107,326)	184.3 (88.8–376.3)	147,793 (71,252–303,928)	212.9 (102.7–441.1)	15.5 (11.6 to 19.8)
Iran (Islamic Republic of)	50,356 (24,041–101,206)	186.6 (89.2–376.7)	175,263 (83,503–351,986)	215.1 (102.5–433.5)	15.2 (13.7 to 17)
Iraq	15,833 (7530–31,775)	196.1 (93.5–394.8)	54,964 (26,620–111,029)	211.7 (102.8–427)	7.9 (4.2 to 11.6)
Jordan	2886 (1381–5762)	201.6 (97.2–405.8)	19,359 (9214–38,956)	230.6 (110.6–463.1)	14.4 (10.7 to 18.5)
Kuwait	1529 (730–3069)	215.9 (102.8–440.5)	8875 (4217–17,985)	238.4 (113.7–478)	10.4 (6.4 to 14.2)
Lebanon	4298 (2051–8673)	191.3 (91.4–385.4)	13,309 (6395–26,581)	227.1 (109.5–453.9)	18.7 (15 to 23.2)
Libya	3967 (1873–8055)	202.9 (96.3–412.7)	13,141 (6298–26,535)	225.2 (107.8–453.3)	11 (7.6 to 14.4)
Morocco	27,325 (13,116–55,792)	189 (90.9–386.4)	74,164 (35,516–151,542)	207 (99.3–422.8)	9.5 (5.8 to 13.8)
Oman	1409 (677–2859)	190.1 (91.8–387.4)	5753 (2747–11,540)	229.5 (109.9–462.6)	20.7 (16.7 to 25)
Palestine	1644 (797–3290)	187.5 (90.9–375.8)	5968 (2849–12,077)	214.8 (102.8–438.1)	14.6 (10.2 to 19.3)
Qatar	346 (164–699)	215.5 (103.4–433.9)	3732 (1782–7558)	236.3 (114.2–472.9)	9.7 (5.9 to 14.1)
Saudi Arabia	12,217 (5871–24,414)	193.2 (93.3–388.7)	57,367 (27,050–114,815)	232.6 (112.2–467.7)	20.4 (16.2 to 24.7)
Sudan	14,804 (7071–29,773)	156.9 (75.3–315.2)	41,062 (19,774–82,321)	195.2 (93.8–392.7)	24.4 (19 to 29.6)
Syrian Arab Republic	9840 (4763–19,631)	181.9 (87.4–364.9)	30,701 (14,735–60,902)	213 (102.3–423.5)	17.1 (13 to 21.7)
Tunisia	9675 (4594–19,643)	188.3 (89.5–382.1)	30,167 (14,549–61,098)	219.9 (106.3–446.5)	16.8 (12.8 to 21)
Turkiye	67,007 (32,366–134,315)	186.9 (89.9–377.7)	217,555 (104,525–438,713)	225.4 (108.3–455.7)	20.6 (16 to 25.2)
United Arab Emirates	1211 (574–2458)	200.8 (95.7–407.3)	17,356 (8125–34,939)	220.6 (104.6–444.4)	9.8 (6.2 to 13.5)
Yemen	7635 (3682–15,593)	151.5 (72.9–310.1)	27,348 (13,109–55,053)	181.3 (87.4–367.3)	19.7 (15.2 to 24.6)
South Asia	1,094,410 (528,656–2,197,896)	181.9 (87.8–368.2)	3,311,236 (1,583,934–6,656,919)	216.9 (104–438)	19.2 (17.3 to 21.3)
Bangladesh	83,619 (40,288–168,313)	172.1 (82.7–347.3)	284,516 (137,093–577,735)	199.7 (96.2–406.2)	16 (12 to 20.1)
Bhutan	453 (220–909)	176.7 (85.3–357.6)	1282 (621–2601)	205.3 (99.4–417)	16.2 (12.2 to 20.5)
India	897,471 (433,854–1,802,636)	185 (89.5–374.8)	2,718,457 (1,302,655–5,462,600)	221.2 (106.3–446.6)	19.5 (17.5 to 21.7)
Nepal	15,939 (7780–32,321)	161.6 (78.7–329.5)	45,782 (22,136–92,352)	190.4 (92.2–385.9)	17.9 (13.9 to 22.4)
Pakistan	96,929 (46,087–195,297)	168.5 (79.9–341.1)	261,200 (124,701–522,199)	200.6 (96–403)	19.1 (16 to 22.5)
East Asia	1,904,132 (915,864–3,832,084)	211 (102.1–424.6)	5,518,037 (2,633,494–11,061,497)	245 (117.4–492.4)	16.1 (13.8 to 18.6)
China	1,829,416 (880,108–3,682,520)	210.6 (101.9–423.9)	5,327,390 (2,541,781–10,678,675)	244.8 (117.3–491.9)	16.2 (13.8 to 18.7)
Democratic People’s Republic of Korea	36,131 (17,172–72,746)	209.3 (99.6–421.8)	77,010 (37,282–155,704)	226 (109.7–457.6)	8 (3.3 to 12.6)
Taiwan	38,585 (18,394–77,246)	234.4 (112–470.1)	113,638 (53,956–229,872)	273.9 (129.6–554.1)	16.8 (12.7 to 20.8)
Oceania	6038 (2899–12,078)	192.3 (92.5–385.9)	17,457 (8450–34,943)	212.9 (103–428.5)	10.7 (8 to 13.5)
American Samoa	61 (29–123)	251.7 (119.4–509.1)	138 (66–282)	270.1 (129.4–551.9)	7.3 (3.8 to 10.8)
Cook Islands	31 (15–61)	239.2 (115.1–469.7)	71 (34–142)	274.3 (131.4–553)	14.7 (10.9 to 18.6)
Fiji	848 (406–1689)	218.2 (103.8–436.7)	2098 (1016–4238)	255.4 (124–518.3)	17.1 (12.8 to 21.3)
Guam	202 (97–405)	243.7 (117.2–490.6)	577 (278–1150)	272.6 (130.8–543.3)	11.9 (8.3 to 15.9)
Kiribati	85 (41–171)	223.8 (107.4–446.5)	191 (91–379)	245.2 (117.4–491.3)	9.6 (6.1 to 13.3)
Marshall Islands	36 (17–73)	219.3 (104.7–440.3)	95 (45–191)	244.9 (116–495.3)	11.6 (8.3 to 15)
Micronesia (Federated States of)	107 (51–214)	219.4 (104.2–438.9)	201 (96–401)	250.5 (119.7–500.9)	14.2 (10.6 to 18.3)
Nauru	11 (5–22)	224.7 (106.7–447.4)	16 (8–32)	257.7 (123–512.7)	14.7 (10.8 to 19.3)
Niue	5 (2–10)	237.4 (113.6–477.3)	6 (3–12)	268.1 (129.4–536.3)	12.9 (9 to 17.4)
Northern Mariana Islands	51 (24–103)	244 (117–494.3)	154 (73–308)	263.6 (125–531.3)	8 (4.4 to 12.1)
Palau	24 (11–49)	238.5 (113.7–486.3)	67 (32–133)	264.8 (126.3–530.3)	11 (7.3 to 14.8)
Papua New Guinea	3445 (1655–6904)	178.2 (85.9–357.9)	11,205 (5417–22,417)	197.9 (96–398.6)	11 (6.9 to 15.2)
Samoa	197 (94–390)	224.8 (107.8–446.4)	380 (182–754)	252 (121–504.6)	12.1 (8.3 to 16.2)
Solomon Islands	283 (135–567)	194.6 (93.4–392.8)	849 (404–1696)	225.6 (107.1–452.5)	16 (11.9 to 20.2)
Tokelau	3 (1–6)	216 (104.3–435.2)	4 (2–8)	255.5 (122.7–515.2)	18.3 (14.2 to 22.9)
Tonga	126 (60–254)	220.3 (105.4–444.7)	203 (98–408)	247.5 (119.1–497.8)	12.4 (8.3 to 16.7)
Tuvalu	15 (7–30)	215 (102.7–438.9)	27 (13–55)	250.6 (118.9–512.6)	16.5 (12.2 to 20.6)
Vanuatu	123 (60–249)	185 (89.1–372.8)	392 (190–791)	209.1 (101.1–422.7)	13 (9.5 to 17.1)
Southeast Asia	432,058 (206,865–871,588)	163.3 (78.1–329.6)	1,357,628 (645,150–2,713,214)	196.2 (93.6–393.4)	20.1 (17.8 to 22.5)
Cambodia	6905 (3332–14,077)	147.3 (71.4–297.5)	22,485 (10,740–45,064)	172.5 (82.6–347.1)	17.1 (12.5 to 22.1)
Indonesia	170,424 (81,687–343,678)	166.2 (79.4–335.2)	516,616 (245,938–1,031,741)	199.4 (95.4–399.4)	20 (17.3 to 22.7)
Lao People’s Democratic Republic	3222 (1551–6532)	151.6 (72.6–307)	8799 (4205–17,778)	177.5 (84.6–358.3)	17.1 (12.2 to 22.5)
Malaysia	18,052 (8537–36,196)	185 (87.6–370.7)	64,150 (30,558–130,651)	217.7 (103.9–443.5)	17.7 (13.9 to 21.7)
Maldives	172 (82–347)	177.6 (84.7–359.5)	821 (389–1649)	213.8 (102–429.9)	20.4 (16 to 24.5)
Mauritius	1445 (685–2923)	193.7 (92.3–391.8)	4164 (1992–8329)	222.3 (106.1–445.2)	14.7 (10.9 to 18.5)
Myanmar	36,583 (17,526–74,378)	154.5 (74.3–312.5)	96,779 (46,238–192,237)	189 (90.5–376.1)	22.3 (17.1 to 27.3)
Philippines	51,853 (24,542–104,045)	168.3 (80–338.4)	169,673 (80,661–339,753)	197.2 (93.9–396.2)	17.2 (15.7 to 18.8)
Seychelles	108 (51–215)	194.2 (92.4–387.6)	279 (133–560)	224.7 (107.3–452.8)	15.7 (11.1 to 20.2)
Sri Lanka	18,854 (8981–38,305)	167.2 (79.9–337.5)	54,025 (25,576–108,719)	196.5 (93.2–395)	17.5 (13 to 21.9)
Thailand	62,734 (30,601–126,180)	166.6 (81.4–337.4)	229,188 (110,606–460,753)	207.8 (100–418.9)	24.7 (19.5 to 30)
Timor-Leste	466 (224–941)	148.8 (71.5–300.8)	1540 (733–3120)	177 (84.2–358.2)	18.9 (13.8 to 24.7)
Viet Nam	60,615 (29,205–120,770)	151.4 (73.1–302)	187,215 (89,044–373,119)	179 (85.1–356.8)	18.2 (12.9 to 23.8)
Central Sub-Saharan Africa	44,741 (21,342–90,400)	191.4 (91.1–387.3)	120,682 (57,012–241,714)	204.3 (97.8–412.2)	6.8 (4.2 to 9.3)
Angola	8723 (4124–17,531)	212.5 (101.5–426)	30,158 (14,453–60,592)	234.9 (113.6–474.4)	10.5 (6.9 to 14)
Central African Republic	2080 (1000–4213)	176.3 (84.7–356.6)	4378 (2112–8874)	183.5 (89.2–370.8)	4.1 (0.6 to 7.7)
Congo	2255 (1071–4563)	207.2 (99–419.2)	6792 (3215–13,707)	228.9 (108.5–462.3)	10.5 (6.9 to 14)
Democratic Republic of the Congo	30,223 (14,461–61,245)	186 (89–379)	75,437 (35,464–151,454)	192.6 (91.3–388.6)	3.5 (0.1 to 7)
Equatorial Guinea	336 (164–682)	168.7 (82.7–341.9)	1308 (623–2612)	236.5 (113.2–474.1)	40.2 (33.7 to 46.7)
Gabon	1125 (539–2295)	196.7 (94.4–402.3)	2609 (1241–5238)	233.5 (111.5–471.9)	18.7 (14.5 to 23.1)
Eastern Sub-Saharan Africa	132,919 (63,595–267,611)	173.5 (83.3–351.4)	358,151 (171,684–719,704)	201 (96.1–405.3)	15.8 (13.6 to 18)
Burundi	3955 (1913–7940)	169.2 (82.5–338.5)	9035 (4359–18,146)	174.1 (84.3–350.3)	2.9 (-0.1 to 6.5)
Comoros	353 (170–715)	173.8 (83.8–351.3)	1012 (486–2055)	197.5 (95.8–401.9)	13.7 (9.5 to 17.6)
Djibouti	259 (124–522)	174.9 (84.2–352.1)	1513 (720–3030)	211.9 (101.2–426.9)	21.2 (16.4 to 25.8)
Eritrea	1955 (934–3916)	162.8 (77.4–326.4)	5431 (2572–10,769)	182 (87.1–363.6)	11.8 (7.3 to 16.2)
Ethiopia	35,252 (16,935–71,268)	174.2 (83.9–354.2)	100,012 (47,612–199,997)	221.1 (106–443.6)	26.9 (21.6 to 31.5)
Kenya	16,150 (7728–32,613)	192.1 (92.4–388.2)	55,737 (26,430–112,103)	229.7 (109.4–462.8)	19.6 (17.3 to 22.1)
Madagascar	8360 (4040–16,918)	161.8 (78.3–325.1)	21,116 (10,250–42,216)	172.4 (82.8–346.8)	6.5 (2.9 to 10.2)
Malawi	6653 (3212–13,377)	168.7 (81.5–339.6)	14,742 (7111–29,707)	189.7 (91.8–382.1)	12.5 (8.5 to 16.6)
Mozambique	10,167 (4849–20,529)	163.4 (78.3–330.2)	21,569 (10,342–43,534)	183.2 (87.9–371)	12.1 (7.9 to 16.2)
Rwanda	4712 (2267–9558)	164.2 (78.8–330.1)	12,116 (5847–24,410)	181 (87.5–364.5)	10.2 (6 to 13.8)
Somalia	4310 (2105–8657)	167.5 (80.8–341)	11,523 (5543–23,310)	175.1 (84.2–352.3)	4.5 (1 to 8.2)
South Sudan	4165 (2025–8314)	161.5 (78.5–322.4)	7379 (3595–14,772)	173.7 (83.9–349.8)	7.6 (3.7 to 11.6)
Uganda	10,808 (5231–21,752)	164.4 (79.5–331.2)	28,901 (13,960–58,727)	185 (89.6–375.2)	12.5 (8.6 to 16.9)
United Republic of Tanzania	20,257 (9681–40,380)	182.4 (87.4–365.7)	53,091 (25,272–107,595)	196.2 (93.8–397)	7.5 (4.5 to 11.3)
Zambia	5468 (2599–11,123)	185.2 (87.9–375.2)	146,62 (7052–29,520)	196.4 (94.6–394.7)	6 (2.7 to 10)
Southern Sub-Saharan Africa	62,841 (30,077–125,999)	229.2 (110.1–461)	148,991 (71,571–298,108)	249.5 (120.4–500.6)	8.9 (7.5 to 10.4)
Botswana	1156 (548–2324)	201.5 (96.2–406.9)	3800 (1831–7655)	241.1 (116.4–485.7)	19.7 (15.8 to 24)
Eswatini	614 (292–1242)	205.2 (97.3–414.2)	1393 (670–2794)	236.7 (114.3–476.4)	15.4 (11 to 20.2)
Lesotho	1603 (775–3194)	187.3 (90.6–373.8)	2455 (1172–4981)	222.4 (106.7–451.5)	18.7 (13.7 to 23.7)
Namibia	1251 (610–2524)	189.2 (92.3–383.1)	3149 (1497–6319)	217.7 (104–438.9)	15 (10.8 to 19.3)
South Africa	50,502 (24,158–101,375)	241.8 (116.1–486.7)	124,233 (59,849–248,722)	259.7 (125.6–521.3)	7.4 (6 to 9)
Zimbabwe	7714 (3734–15,456)	184.6 (89–371.2)	13,961 (6715–27,908)	191 (92.2–381.9)	3.5 (-0.2 to 7.5)
Western Sub-Saharan Africa	168,735 (81,206–339,506)	187.5 (90.3–378.8)	442,087 (212,439–888,884)	210.1 (101.1–424.7)	12.1 (10.9 to 13.2)
Benin	3619 (1743–7290)	180.3 (86.8–363.8)	11,870 (5698–23,987)	216.4 (103.7–435.1)	20.1 (15.8 to 24.5)
Burkina Faso	7332 (3537–14,818)	165.6 (80.1–334.8)	17,798 (8612–35,905)	183.5 (89.3–369)	10.8 (6.8 to 14.8)
Cabo Verde	405 (196–813)	184.2 (89.1–371.4)	1027 (494–2071)	222.3 (106.7–450)	20.7 (15.9 to 25.3)
Cameroon	8831 (4272–17,831)	190.4 (92.1–383.3)	29,381 (14,159–59,080)	217.4 (105.2–440)	14.2 (10.2 to 18.4)
Chad	4708 (2276–9519)	166.6 (81–337.1)	10,825 (5277–21,891)	176.4 (85.8–356)	5.9 (1.9 to 9.7)
Côte d'Ivoire	7781 (3776–15,610)	181 (87.7–366.6)	25,228 (12,158–51,056)	206.6 (99–421.1)	14.1 (10.3 to 18.1)
Gambia	664 (321–1334)	183.2 (88.2–367.5)	2215 (1061–4490)	217 (104.7–440.4)	18.4 (13.8 to 22.6)
Ghana	14,300 (6761–28,809)	219.4 (104.4–443)	41,273 (19,681–82,750)	230.9 (110.7–463.5)	5.3 (1.5 to 9.8)
Guinea	5700 (2768–11,612)	170.4 (82.6–346)	10,941 (5275–21,931)	187.2 (90.6–375)	9.9 (6.2 to 13.8)
Guinea-Bissau	705 (345–1426)	174.4 (85.6–351.7)	1498 (716–2995)	192.9 (92.6–389)	10.6 (6.8 to 14.6)
Liberia	2100 (1009–4194)	181.4 (87.4–362.6)	4885 (2359–9922)	208.1 (100.4–423.1)	14.7 (11.1 to 18.4)
Mali	6952 (3321–14,004)	168.9 (80.9–338.2)	17,679 (8526–35,194)	189.6 (91.9–381.3)	12.3 (7.9 to 16.8)
Mauritania	1921 (932–3910)	190.2 (92.2–388)	4959 (2363–10,023)	220.1 (105.3–443.8)	15.7 (11.7 to 19.8)
Niger	4876 (2377–9773)	167.3 (81.7–336.7)	15,514 (7468–31,212)	178.3 (86.4–355)	6.6 (2.7 to 10.5)
Nigeria	86,775 (41,755–174,520)	192.4 (92.6–388.4)	213,566 (102,290–428,807)	217.1 (104.5–439.4)	12.8 (11.3 to 14.3)
Sao Tome and Principe	127 (61–262)	196.5 (94.7–403.5)	287 (138–580)	238.2 (114.6–481.4)	21.3 (16.9 to 26.6)
Senegal	6091 (2937–12,334)	184.6 (88.7–373.2)	16,773 (8031–33,485)	206.5 (98.6–415.1)	11.9 (8.1 to 15.8)
Sierra Leone	3538 (1709–7084)	171.9 (83.5–344.8)	7830 (3753–15,712)	197.2 (94.6–398.3)	14.7 (10.3 to 19.2)
Togo	2304 (1110–4594)	178.5 (85.9–356.5)	8532 (4036–17,272)	205.5 (98–413.4)	15.1 (11 to 19.9)

DALYs = disability-adjusted life years, UI = uncertainty intervals.

### 3.4. Age and sex patterns

**Figure 4.**
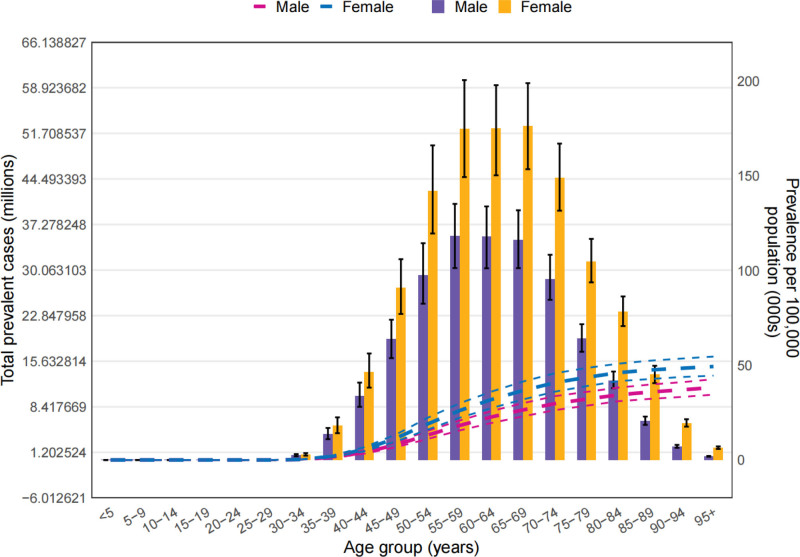
Global prevalence and case numbers of osteoarthritis by age and gender, 2021.

**Figure 5.**
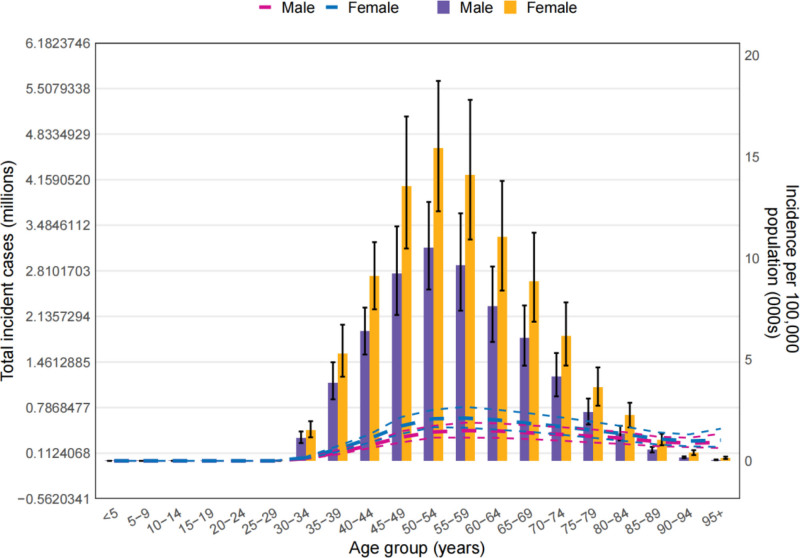
Global number of incident cases and incidence of osteoarthritis by age and sex, 2021.

**Figure 6.**
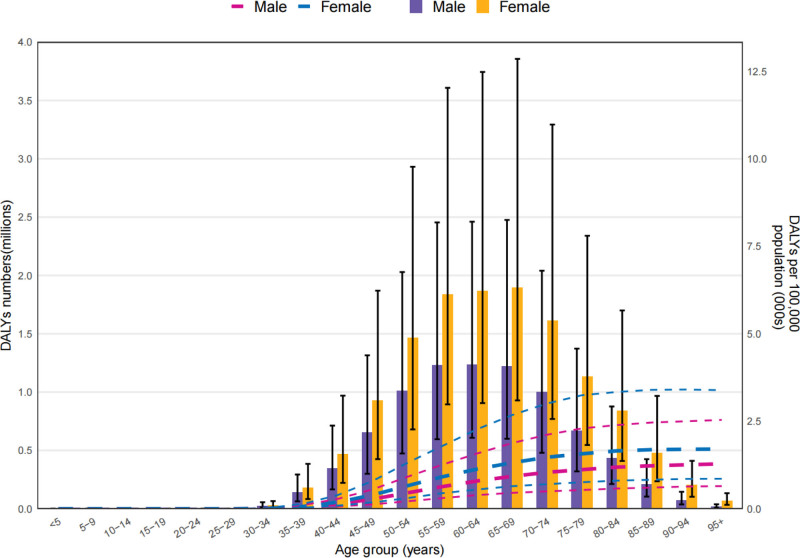
Global number of DALYs cases and DALYs rates of osteoarthritis by age and sex, 2021 burden of osteoarthritis by Sociodemographic Index. DALYs = disability-adjusted life years.

In 2021, the prevalence rates were notably higher among females and escalated with increasing age, reaching their zenith in the age group over 95 years for both genders. Additionally, the count of prevalent cases rose with age, peaking in the 65 to 69 years age bracket for both men and women. Subsequently, a downward trend in the number of prevalent cases was observed extending to the oldest age group. The prevalence figures for the 3 groups aged 55 to 69 years were quite comparable (as depicted in Fig. 4). The global incidence rate was predominantly higher among women and exhibited an upward trend with increasing age, peaking in the 50 to 54 years age bracket. The highest number of new cases was observed in the 55 to 59 years age group, after which a decline was noted, continuing through to the oldest age group (Fig. 5). Figure 6 illustrates the global number of DALYs cases and DALY rates for osteoarthritis per 100,000 populations, categorized by age and sex. The counts and rates for males and females do not significantly differ from the patterns observed in prevalence and incidence structures. Both genders exhibit a gradual increase in DALYs as age progresses.

### 3.5. Burden of osteoarthritis by SDI

**Figure 7.**
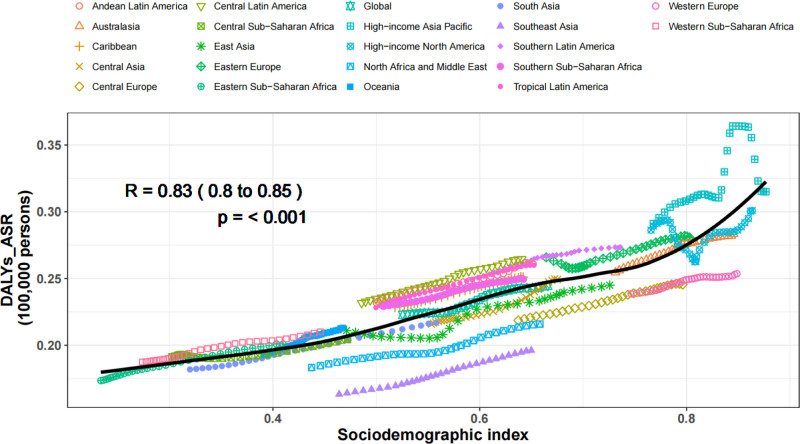
Age-standardized DALY rates for osteoarthritis for 21 Global Burden of Disease (GBD) regions by SDI, 1990 to 2021. DALYs = disability-adjusted life years, SDI = Sociodemographic Index.

**Figure 8.**
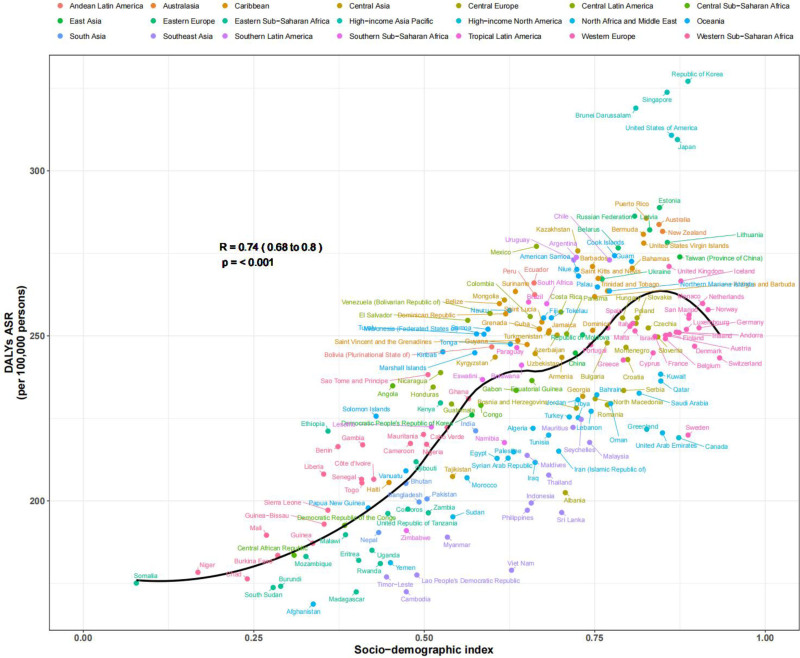
Age-standardized DALY rates of osteoarthritis by 204 countries and territories and SDI index. DALYs = disability-adjusted life years, SDI = Sociodemographic Index.

Globally and across all GBD regions, the age-standardized DALY rate was positively correlated with the SDI during the period from 1990 to 2021 (Fig. 7). The correlation coefficient (*R*) was 0.83 (95% confidence interval: 0.80–0.85), and the correlation was statistically significant (*P* < .001). At the global scale, as well as within the regions of Central Latin America, Tropical Latin America, Australasia, Andean Latin America, Central Sub-Saharan Africa, Southern Latin America, and Oceania, the observed estimates of the burden of osteoarthritis exceeded the levels anticipated based on the SDI from 1990 to 2021. In 2021, a positive correlation between the age-standardized DALY rate of osteoarthritis and SDI was observed across 204 countries and territories (Fig. 8). The correlation coefficient (*R*) was 0.74 (95% confidence interval: 0.68–0.80), and the correlation was statistically significant (*P* < .001). The age-standardized DALY rate was higher than the expected level for a number of countries/territories, including the United States of America, Singapore, Japan, and the Republic of Korea. Conversely, in several other countries, such as Viet Nam, Afghanistan, and Sudan, the age-standardized DALY rate was lower than anticipated.

## 4. Discussion

This epidemiological assessment presents contemporary estimations of osteoarthritis burden across 204 nations and territories from 1990 to 2021, examining prevalence metrics, incidence rates, and DALYs. The analysis reveals that osteoarthritis imposed a substantial global health burden in 2021, with surveillance systems documenting 606.9 million existing cases worldwide, 46.6 million newly recorded diagnoses, and approximately 21.3 million DALYs measuring cumulative health deterioration. This study incorporated and analyzed the 2021 global burden of osteoarthritis data from the GBD database, providing previously unreported national-level estimates. Previous studies have reported epidemiological estimates of osteoarthritis across GBD regions using data from the GBD 2019 Study.^[22,23]^ However, due to inherent biases in analytical indicators and methodological disparities, these earlier findings cannot fully encompass or be directly compared with our current analysis. Previous studies did not report the age-standardized DALYs rate of osteoarthritis or its association with the SDI. Additionally, the reported geographic coverage was limited.^[16,24–28]^

From 1990 to 2021, there has been a noted upward trend in both the prevalence and incidence of osteoarthritis. This is consistent with results from earlier research,^[29]^ suggesting that osteoarthritis is on track to become a major contributor to global mortality.^[24]^ Consequently, there is a pressing need to focus on the prevention, management, and treatment strategies for this condition. The condition’s increasing prevalence is particularly notable in high-income regions of the Asia Pacific, North America, Australasia, and Southern Latin America.

As both this study and previous ones^[16,23,24]^ have shown, older women are disproportionately affected by osteoarthritis. Thus, it is crucial that targeted prevention, management, and treatment efforts are directed towards this demographic. Globally, the prevalence rates for women are higher and they escalate with age, with the oldest age groups experiencing the highest rates for both genders in 2021. This trend is in line with findings from prior studies using data from the GBD 2020.^[16]^ While end-stage osteoarthritis can be managed through various treatments, there is currently no cure for the condition.

It has been observed that the burden of osteoarthritis is positively correlated with the SDI level. Countries with a high-SDI level tend to experience an exceptionally high burden of osteoarthritis. This high burden is not only evident in developed countries such as Japan and Singapore but also in countries like Estonia that are undergoing development.

Osteoarthritis is a significant cause of pain, disability, and socioeconomic costs globally. While treatments for osteoarthritis include both conservative and surgical approaches, preventing its progression is one of the most crucial strategies. Key modifiable risk factors that should be targeted include overweight or obesity, joint injuries, and smoking.^[30,31]^ These factors are potentially preventable. Maintaining a healthy body mass index is crucial in the fight against osteoarthritis, and it should be a key focus in worldwide, regional, and national efforts aimed at preventing, managing, and treating this condition.

### 4.1. Strengths and limitations

This study seeks to provide an extensive analysis of the impact of osteoarthritis through 2021, considering worldwide, regional, and national data. It offers significant insights for future research on osteoarthritis and the development of strategies for its prevention and management. We have carefully documented various elements linked to the occurrence and impact of osteoarthritis, measured by DALY, considering variables such as gender, age, and the SDI. By utilizing the comprehensive dataset from the GBD 2021, our research includes a thorough examination of all joints affected by osteoarthritis, including the hip, knee, hand, and additional areas, thus broadening the scope of our findings.

However, this study has several limitations that should be acknowledged. While a number of countries and regions contributed data to the GBD 2021, the overall data coverage remains limited. Due to the constraints of database completeness, this study does not investigate the relationship between osteoarthritis and years lived with disability. Moreover, the study does not conduct a detailed analysis of risk factor attribution for osteoarthritis, such as lifestyle habits and disease awareness.

## 5. Conclusion

Global osteoarthritis burden continues to rise, with older women and high-SDI countries most affected. Prevention remains feasible: avoiding joint injury, maintaining a healthy weight, and implementing age-friendly physical-activity and workplace policies are immediate, low-cost levers for mitigation.

Although this GBD 2021 analysis covers 204 countries and territories with the most current evidence available, gaps persist in low- and middle-income settings and joint-specific risk attribution is still lacking. Future studies should integrate prospective cohorts with rich lifestyle and biomarker data, develop joint-specific projection models that account for obesity trends, occupational exposure, and population aging, and evaluate the cost-effectiveness of community-level prevention programs. Turning today’s burden into tomorrow’s prevention strategy will depend on strengthening this evidence base.

## Acknowledgments

We acknowledge with gratitude the Institute for Health Metrics and Evaluation staff and collaborators for their invaluable efforts in preparing these publicly accessible datasets.

## Author contributions

**Conceptualization:** Shu-Feng Ye.

**Data curation:** Shu-Feng Ye.

**Formal analysis:** Shu-Feng Ye.

**Methodology:** Wei-Qi Liu, Zi-Liang Chen.

**Software:** Wei-Qi Liu.

**Supervision:** Yao-Hui Zhou.

**Validation:** Yao-Hui Zhou.

**Visualization:** Tao Zeng.

**Writing – original draft:** Tao Zeng.

**Writing – review & editing:** Zi-Liang Chen
